# Metabolomics 2023 workshop report: moving toward consensus on best QA/QC practices in LC–MS-based untargeted metabolomics

**DOI:** 10.1007/s11306-024-02135-w

**Published:** 2024-07-09

**Authors:** Jonathan D. Mosley, Warwick B. Dunn, Julia Kuligowski, Matthew R. Lewis, María Eugenia Monge, Candice Ulmer Holland, Dajana Vuckovic, Krista A. Zanetti, Tracey B. Schock, the Metabolomics Quality Assurance, Quality Control Consortium (mQACC)

**Affiliations:** 1 Center for Environmental Measurement and Modeling, Environmental Protection Agency, Athens, GA USA; 2 Centre for Metabolomics Research, Department of Biochemistry and Systems Biology, Institute of Systems, Molecular and Integrative Biology, University of Liverpool, Biosciences Building, Crown Street, Liverpool, L69 7ZB UK; 3 Neonatal Research Group, Health Research Institute La Fe, Avda. Fernando Abril Martorell 106, 46026 Valencia, Spain; 4 Bruker Life Sciences Mass Spectrometry, Bruker UK Ltd., Welland House, Longwood Close, Coventry, CV4 8AE UK; 5 National Phenome Centre & Division of Systems Medicine, Department of Metabolism, Digestion & Reproduction, Imperial College London, Hammersmith Campus, London, W12 0NN UK; 6 Centro de Investigaciones en Bionanociencias (CIBION), Consejo Nacional de Investigaciones Científicas y Técnicas (CONICET), Godoy Cruz 2390, C1425FQD Ciudad de Buenos Aires, Argentina; 7 Eastern Laboratory, Office of Public Health Science (OPHS), Food Safety and Inspection Service (FSIS), U.S. Department of Agriculture (USDA), Athens, GA 30605 USA; 8 Department of Chemistry and Biochemistry, Concordia University, 7141 Sherbrooke Street West, Montreal, QC H4B 1R6 Canada; 9 Office of Nutrition Research, Division of Program Coordination, Planning, and Strategic Initiatives, Office of the Director, National Institutes of Health, Bethesda, MD USA; 10 Chemical Sciences Division, National Institute of Standards and Technology (NIST), Charleston, SC 29412 USA

**Keywords:** Metabolomics, Quality control (QC) samples, Liquid chromatography–mass spectrometry (LC–MS), Metabolite annotation, Metabolite identification, Reference materials, Data quality, Quality assurance

## Abstract

**Introduction:**

During the Metabolomics 2023 conference, the Metabolomics Quality Assurance and Quality Control Consortium (mQACC) presented a QA/QC workshop for LC–MS-based untargeted metabolomics.

**Objectives:**

The Best Practices Working Group disseminated recent findings from community forums and discussed aspects to include in a living guidance document.

**Methods:**

Presentations focused on reference materials, data quality review, metabolite identification/annotation and quality assurance.

**Results:**

Live polling results and follow-up discussions offered a broad international perspective on QA/QC practices.

**Conclusions:**

Community input gathered from this workshop series is being used to shape the living guidance document, a continually evolving QA/QC best practices resource for metabolomics researchers.

**Supplementary Information:**

The online version contains supplementary material available at 10.1007/s11306-024-02135-w.

## Introduction

High quality data is a cornerstone of rigor and reproducibility in untargeted metabolomics and can be achieved through standardization and implementation of best quality assurance (QA)/quality control (QC) practices. In an effort to define and promote these, the Metabolomics Quality Assurance and Quality Control Consortium (mQACC, https://www.mqacc.org/) was established in 2017, is currently composed of more than 120 international members (Beger et al., 2019), and is accepting others interested in joining this initiative. The mQACC Best Practices Working Group has actively engaged the metabolomics community over the last 5 years through a combination of 11 virtual and in-person interactive forums and workshops with a total attendance exceeding 600 participants across all events.

The working group previously hosted a workshop attended by more than 200 participants at the 2022 Metabolomic Society conference in Valencia, Spain, to disseminate to the broader metabolomics community findings from discussion of four selected QC topics (Dunn et al., 2023). Those areas were: pooled and intra-study QC samples, system suitability evaluation, use of internal standards, and design of the analytical batch. A follow-up workshop was designed to relay the results from the four topical forums held throughout 2021–2022 to a broader community and solicit further feedback. This report details that workshop, held at Metabolomics 2023 in Niagara Falls, Canada. The aims of the 2023 workshop were to (1) disseminate findings from the mQACC Best Practices Working Group’s extensive community engagement efforts to establish best practices for LC–MS data collection in untargeted metabolomics; and (2) solicit additional feedback from the international metabolomics community on the compiled and summarized findings to establish an open-access best practices living guidance document that will be freely accessible to researchers and will evolve over time. The objective of this workshop report is to disseminate findings obtained through this community engagement process and further engage scientists in the field on QA/QC best practices in metabolomics.

## Workshop structure

**Fig. 1:**
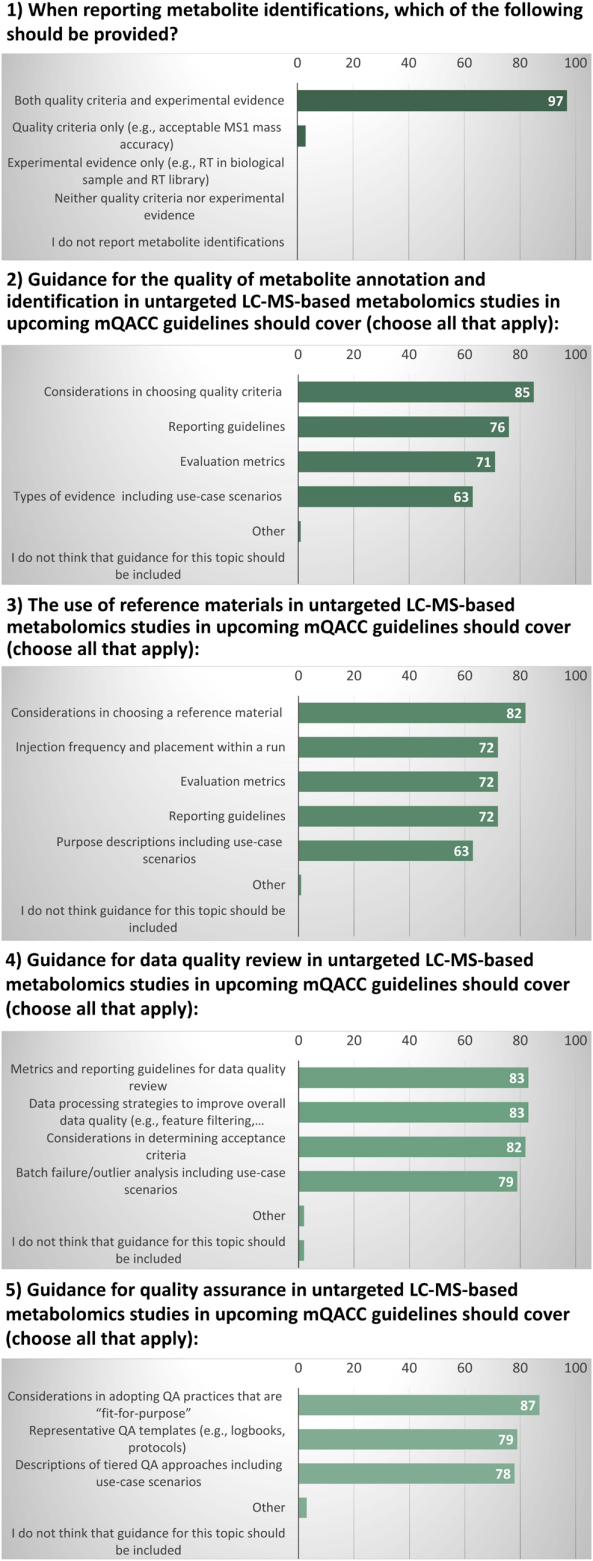
Polling questions to survey attendees during the workshop using the on-line conference app, EventsAIR, and their responses (N = 62–68). Note: All questions except for the first were ‘choose all that apply’ questions

Similar to the 2022 workshop, the 2023 workshop was structured to disseminate the latest information gathered on four selected QA/QC topics through the mQACC Best Practices Working Group community engagement activities and further extend discussions on these topics to encourage community adoption and continued evolution of metabolomics QA/QC best practices (Dunn et al., 2023). The four key topics chosen for 2023 were: (1) the quality of metabolite identification/annotation; (2) the use of reference materials (RM); (3) data quality review; and (4) quality assurance. The workshop was conducted by six mQACC members during a 2-h session and designed to be interactive between the organizers and attendees. After a 5-min introduction to mQACC, the main part of the workshop consisted of four 25-min sessions covering the four key QA/QC areas. Each session consisted of a presentation disseminating the findings from three Best Practice Working Group forums and an internal survey of mQACC members. Each presentation was followed by live polling for the audience managed through the conference’s mobile application, EventsAIR. The polls were recorded, and the results are provided in Fig. 1. After polling, the audience was further engaged with facilitated discussion to gather additional insight from the workshop attendees on each topic. This structure was designed to garner community feedback and build awareness of how the feedback will be used to support QA/QC best practices for untargeted LC–MS-based metabolomics.

## Workshop content

The majority of the presented content comprised the findings from recent Best Practice Working Group efforts covering the four QA/QC focus areas. In addition to recent findings, the attendees were surveyed during the workshop using five live polling questions to solicit additional feedback from audience members. The full results accompany this report (see Supplemental File 1), which include responses to polling questions for each of the first three topics and the results of an internal survey of mQACC members regarding quality assurance. The major findings and discussion points are presented below.

The first presentation on quality of metabolite identification/annotation covered the types of data used to identify metabolites (e.g., MS^1^ spectra and retention time (RT) data) and the quality indicators used to assess confidence in metabolite identification (e.g., *m/z* and RT error). Types of libraries used for identification may include authentic chemical standards or in-silico generated libraries for MS/MS and RT. Searching multiple types of libraries, such as in-house and publicly available options, provides supporting evidence and maximizes the likelihood of identification. The user defines and reports the criteria used to determine if identification is of an acceptable quality. Many forum poll respondents reported that they identify less than 200 metabolites in a study.

The presentation on the use of RM for untargeted metabolomics focused on well-characterized, homogeneous, stable materials designed for QA/QC practices. Appropriate RM can be composed of biological (involving a matrix) or synthetic materials sourced commercially or created in-house. The mQACC Reference and Test Material Working Group recently prepared a resource of such materials available to the metabolomics community (Lippa et al., 2022). RMs are used both to assess data quality (e.g., repeatability/reproducibility) and correct data quality (e.g., batch correction). An RM may have multiple purposes, for example normalization, harmonization across studies and/or evaluation of intra-study precision. The purpose of an RM will be an important factor defining the pertinent characteristics when selecting a material, such as stability for a long-term RM or traceability to an SI unit for quantitation. Material cost and lack of educational resources may contribute to low RM use in the community; however, applying and reporting RM use in metabolomics studies is encouraged to ensure confidence in generated data.

The data quality review presentation discussed both real-time and post-acquisition phases of an LC–MS-based untargeted metabolomics workflow. A real-time review offers early detection of problems using data quality metrics such as signal response fluctuations and RT drift of QC samples or internal standard response fluctuations across study samples. Post-acquisition review is a holistic assessment of all features/metabolites across sample types that can identify batch effects and evaluate *m/z* accuracy of the entire study, for example. The main goal of post-acquisition review is to accept/reject the data collected prior to further statistical and biological interpretation. Typical acceptance criteria applied during data quality review can include, but are not limited to, evaluating maximum intensity variation expressed as relative standard deviation (%RSD) or tight QC clustering in a principal component analysis scores plot. The need for more objective acceptance criteria than visual inspection was also discussed, such as D ratios. Should a system/sample failure or outlier be identified, corrective actions can be taken like accepting a batch up to the last “good” QC or reanalyzing failed samples. Also, data manipulation strategies (e.g., coefficient of variation and frequency filtering) can help to improve data quality prior to data analysis but appear to be underused by the community. Adoption of redundant quality control strategies employing multiple fit-for-purpose QC sample types, such as pooled QC, phenotypic QC and RM, ensures study quality can be properly evaluated even when one QC sample type fails. This minimizes loss of precious samples or entire studies.

The final presentation concerned quality assurance, or the processes performed independent of data acquisition to instill confidence that quality requirements will be fulfilled (Evans et al., 2020). Quality assurance practices differ across metabolomics research groups with respect to the research environment and institutional type including clinical and academic research labs, core facilities, contract research organizations and highly regulated laboratories (e.g., pharma and government). Thus, to gather initial information on this topic, an internal mQACC survey was a more appropriate method (as opposed to polling questions) to capture the diverse QA practices from a representation of academic, industrial and government laboratories worldwide. This foundational information was used to craft a workshop presentation that would incite a complex discussion eliciting broad community input from the attendees. Results of the internal survey of mQACC members covered fit-for-purpose applications for several QA topics, including audits, documentation, and standard operating procedures (see Supplemental File 1 for full application details). Extent of QA should be fit- for- purpose and appropriately scaled to lab environment and activities. This may include a tiered QA approach ranging from most common practices (e.g., staff training and logbooks) employed across all labs to least common practices (e.g., external quality system audits) employed specifically in regulated environments. In general, adopting simple, enabling QA systems are key for user compliance.

## Attendee engagement

The workshop presenters surveyed the audience with live polling followed by a guided discussion subsequent to each topic presentation (see Fig. 1). Results from the polling show that the workshop participants (≈100) were consistently engaged throughout the 2-h session [number of responses: N = 63 (Question 1); N = 62 (Question 2); N = 68 (Question 3); N = 66 (Question 4); and N = 67 (Question 5)]. Attendees actively communicated with the speakers during the guided discussion portions of the workshop, which constituted a minimum of 35% of the scheduled time. Detailed notes from two separate notetakers indicated that approximately 10–15% of the responders participated in the discussions at least once, and the majority of these individuals participated multiple times. Attendees who participated in the polling, discussion, or both, included representatives from academia, industry, and government and spanned a wide range of career stages. This diversity is important considering the two primary learning outcomes of the workshop, namely that (1) feedback provided by attendees will be used to support documentation and dissemination of QA/QC best practices for untargeted LC–MS-based metabolomics, and (2) the workshop facilitated learning how to participate in mQACC, including mechanisms to contribute to the best practices community engagement efforts. Regarding the former, it is critical to develop and establish best practices that are usable by the widest possible audience of metabolomics users and practitioners. Further, mQACC attributed at least 25% of its growth after the conference to the success of this workshop. Specifically, 4 out of 16 new members who joined after July 1, 2023 as of the time of writing can be traced to workshop participants who were not members of mQACC previously. Undeniably, it is essential to mQACC’s mission to garner representation from the global metabolomics community.

## Future actions

**Fig. 2:**
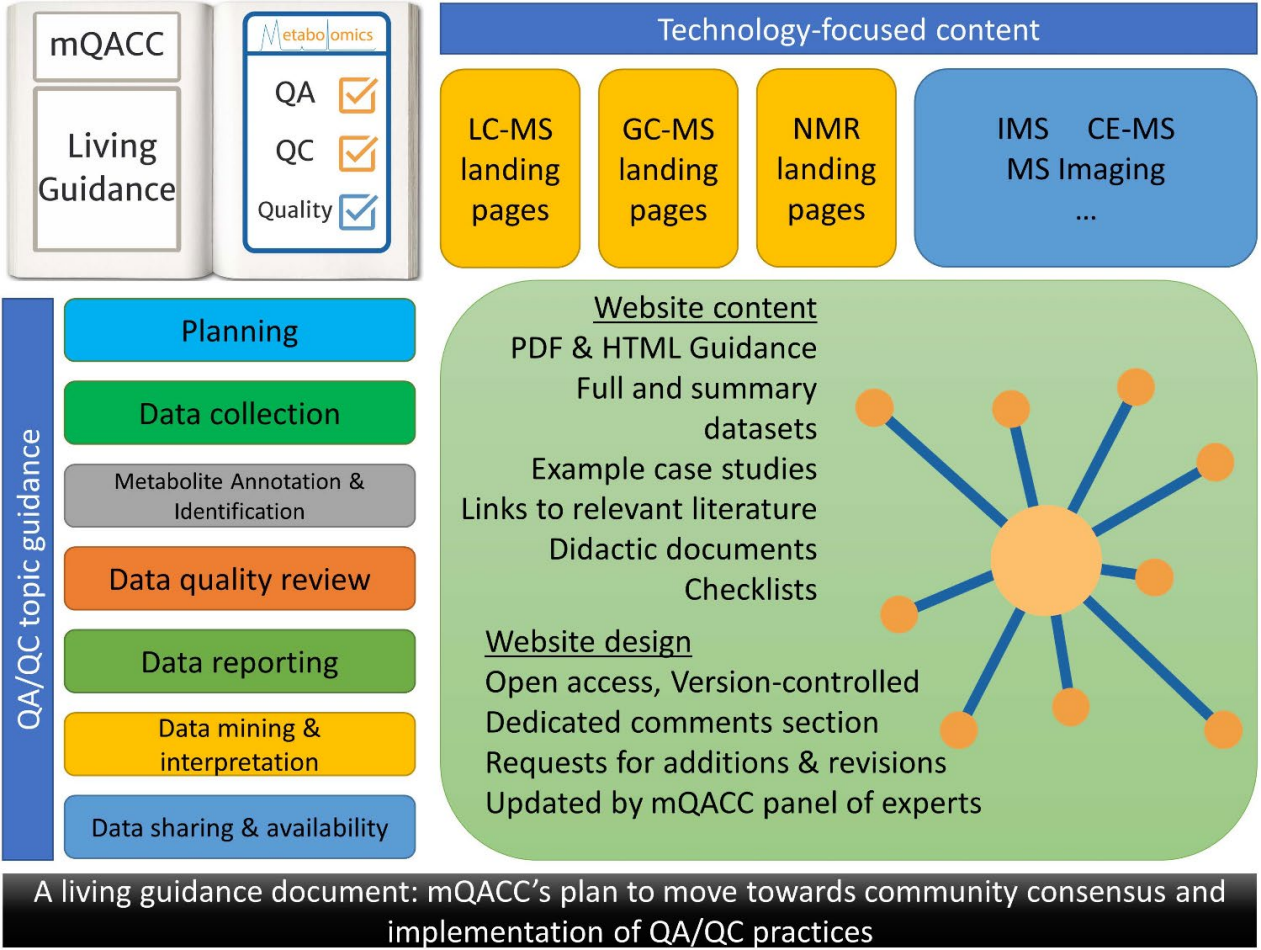
The conceptual format of the mQACC Living Guidance document and its accompanying website as an open-access community resource

The discussions reported here have contributed towards building an open-access best practices living guidance document that reflects the community feedback received. This living document is outlined in Fig. 2. A detailed description of the proposed content of its first version and the framework around which it will be developed and agreed upon has been published (Mosley et al., 2024). As of early 2024, mQACC is designing the website and assembling its content. Once guidance on topics covered in the 2022 and 2023 Metabolomics Society conference workshops are added, an initial version will be disseminated via a publicly accessible website and accompanied by a publication. Furthermore, as mQACC engages the community regarding additional technologies (e.g., gas chromatography-mass spectrometry and nuclear magnetic resonance) and QA/QC topics (data mining and interpretation, data sharing and availability, etc.), additional guidance will be incorporated into the living document, and new versions will be released. Importantly, the website’s content will be presented and discussed in public forums (e.g., at conference workshops and virtual interactive forums) where community participation is paramount. This plan of action will allow for continued critical discussions to further fine tune both the format and the evolving content of the mQACC Living Guidance and encourage wide community adoption of best current practices in untargeted metabolomics.

### Supplementary Information

Below is the link to the electronic supplementary material.

Supplementary file1 (PDF 6328 KB)

## Data Availability

All data are included in the manuscript.

## Declarations

### Disclaimer

This article reflects the views of the author(s) and does not necessarily represent the views or policies of the U.S. Environmental Protection Agency (EPA), the National Institutes of Health (NIH), nor the National Institute of Standards and Technology (NIST). Certain commercial equipment, instruments, trade names, products or materials are identified in this paper in order to specify the experimental procedure adequately. Such identification is not intended to imply or convey and should not be interpreted as conveying official approval, recommendation or endorsement by NIST, the EPA, or the NIH, nor is it intended to imply that the materials or equipment identified are necessarily the best available for the purpose.
