# Supplementary material for: Metabolomics 2023 workshop report: moving toward consensus on best QA/QC practices in LC–MS-based untargeted metabolomics
Source: Metabolomics. 2024 Jul 9;20(4):73. doi: 10.1007/s11306-024-02135-w (PMC11233279; doi:10.1007/s11306-024-02135-w)
Supplement: Supplementary file 1 — Supplementary file1 (PDF 6328 KB) [file 11306_2024_2135_MOESM1_ESM.pdf]

Metabolomics
Quality Assurance & Quality Control Consortium (mQACC)

Moving Toward Consensus

mQACC Community Engagement on Best QA/QC Practices in LC-MS-Based Untargeted Metabolomics

@mQACC

Monday, June 19th
8:15 am – 10:15 am

Disclaimer

The findings and conclusions in this presentation have not been formally disseminated by EPA and should not be construed to represent any Agency determination or policy.

History and early developments

Metabolomics Quality Assurance & Quality Control Consortium (mQACC)

- **mQACC** was formed following a Think Tank meeting at the National Cancer Institute in October 2017
- **Mission**: To engage the metabolomics community to communicate and promote the development, dissemination and harmonization of best QA/QC practices in untargeted metabolomics
- **Membership:** 106 scientists across 4 continents from academia, industry and government organizations

Metabolomics (2019) 15:4
https://doi.org/10.1007/s11306-018-1460-7

SHORT COMMUNICATION

CrossMark

Towards quality assurance and quality control in untargeted metabolomics studies

Richard D. Beger[1] · Warwick B. Dunn[2] · Abbas Bandukwala[3] · Bianca Bethan[4] · David Broadhurst[5] · Clary B. Clish[6] · Surendra Dasari[7] · Leslie Derr[8] · Annie Evans[9] · Steve Fischer[10] · Thomas Flynn[3] · Thomas Hartung[11] · David Herrington[12] · Richard Higashi[13] · Ping-Ching Hsu[14] · Christina Jones[15] · Maureen Kachman[16] · Helen Karuso[17] · Gary Kruppa[18] · Katrice Lippa[15] · Padma Maruvada[19] · Jonathan Mosley[20] · Ioanna Ntai[21] · Claire O'Donovan[22] · Mary Playdon[23] · Daniel Raftery[24] · Daniel Shaughnessy[25] · Amanda Souza[21] · Timothy Spaeder[9] · Barbara Spalholz[23] · Fariba Tayyari[26] · Baljit Ubhi[27] · Mukesh Verma[23] · Tilman Walk[4] · Ian Wilson[28] · Keren Witkin[23] · Daniel W. Bearden[29,30] · Krista A. Zanetti[23]

Received: 15 October 2018 / Accepted: 5 December 2018 / Published online: 3 January 2019
© Springer Science+Business Media, LLC, part of Springer Nature 2019

Abstract
We describe here the agreed upon first development steps and priority objectives of a community engagement effort to address current challenges in quality assurance (QA) and quality control (QC) in untargeted metabolomic studies. This has included (1) a QA and QC questionnaire responded to by the metabolomics community in 2015 which recommended education of the metabolomics community, development of appropriate standard reference materials and providing incentives for laboratories to apply QA and QC; (2) a 2-day 'Think Tank on Quality Assurance and Quality Control for Untargeted Metabolomic Studies' held at the National Cancer Institute's Shady Grove Campus and (3) establishment of the Metabolomics Quality Assurance and Quality Control Consortium (mQACC) to drive forward developments in a coordinated manner.

Keywords Quality assurance (QA) · Quality control (QC) · Community engagement · Test materials · Reporting metrics

What is quality control and quality assurance?

Metabolomics Quality Assurance & Quality Control Consortium (mQACC)

- **Quality Control:** Processes related to the procedures applied **during and after data acquisition**

- **Quality Assurance:** Processes related to the procedures applied in **preparation for data acquisition**

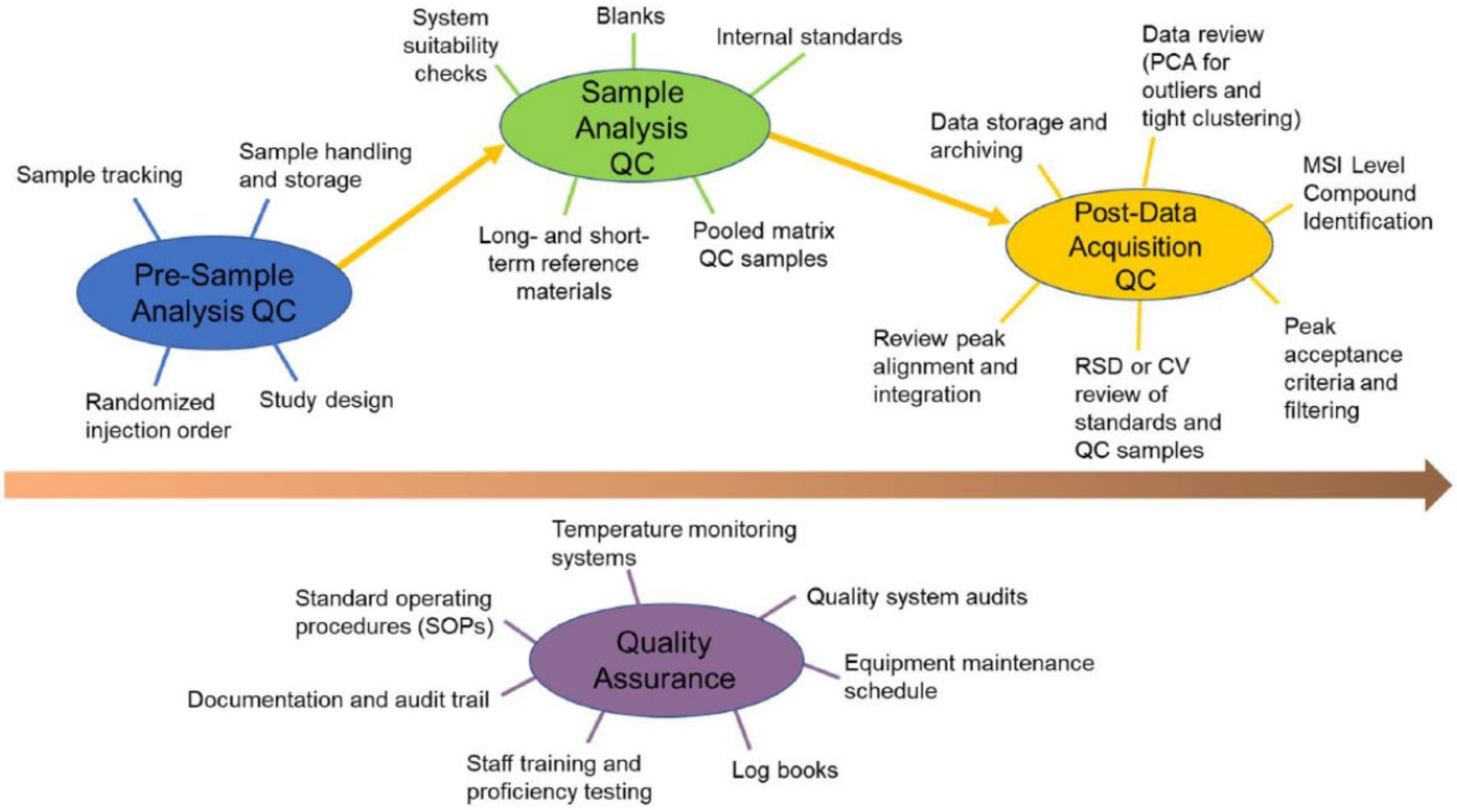

Evans AM, et al. Metabolomics. 2020 Oct 12; 16(10): 113

Workshop objectives

1. To disseminate findings from the mQACC Best Practices Working Group’s extensive community engagement efforts to establish best practices for LC-MS data collection in untargeted metabolomics.

2. To solicit further feedback from the international metabolomics community on the compiled and summarized findings to establish an open-access best practices “living guidance” document that will be freely accessible to researchers.

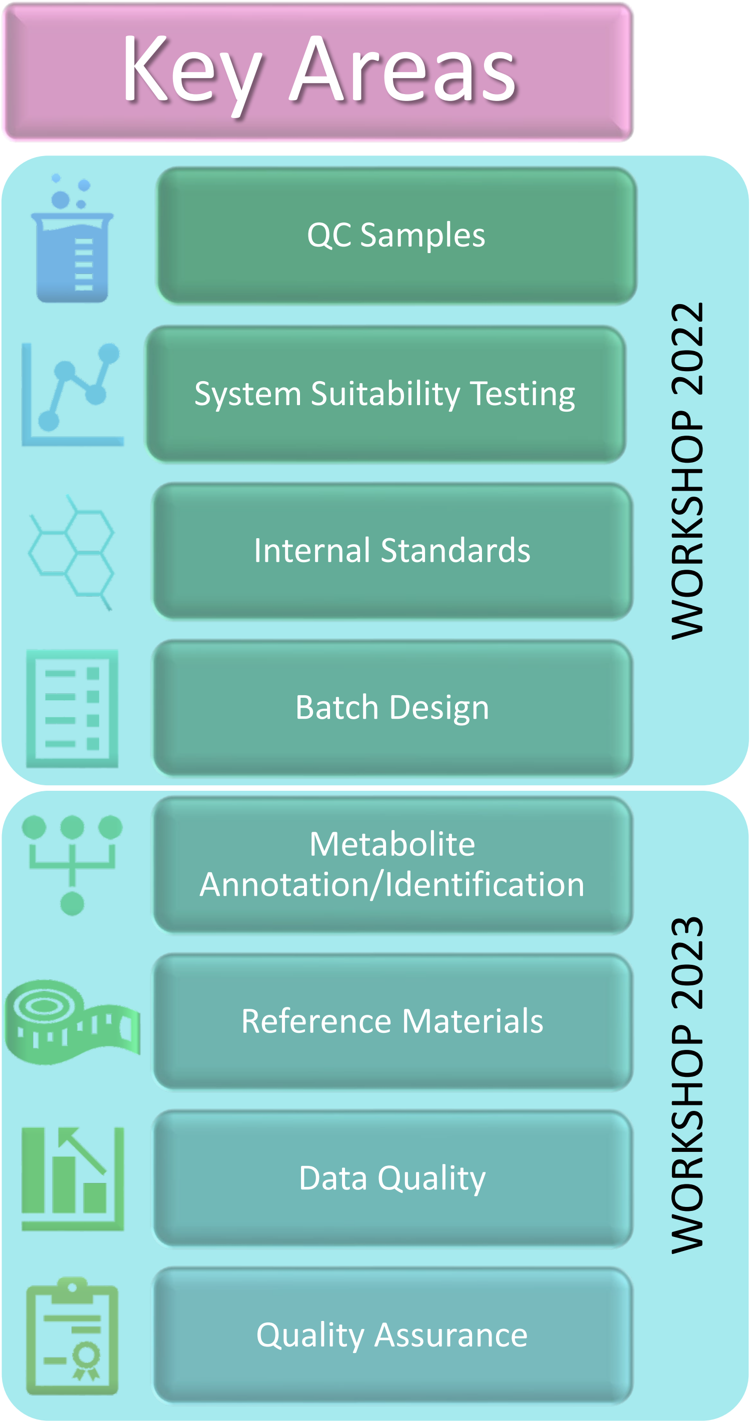

The next 110 minutes

Disseminate findings from the mQACC Best Practices Working Group's extensive community engagement efforts in relation to LC-MS untargeted metabolomics (80 minutes)

- Quality of metabolite identification – Rick Dunn, University of Liverpool, UK
- Use of reference materials – Tracey Schock, National Institute of Standards and Technology, USA
- Data quality review – Dajana Vuckovic, Concordia University, Canada
- Quality assurance – Matt Lewis, Bruker Life Sciences Mass Spectrometry, UK

Discussion on Living Guidance Document - Jonathan Mosley, Environmental Protection Agency, USA (30 minutes)

Acknowledgements

Metabolite Identification

- Rick Dunn
- Matthew Lewis
- Jonathan Mosley
- Claire O'Donovan
- Candice Ulmer
- Dajana Vuckovic
- Krista Zanetti

Reference Materials

- Julia Kuligowski
- Matthew Lewis
- Jonathan Mosley
- Claire O'Donovan
- Dajana Vuckovic
- Krista Zanetti

Data Quality Review

- Helen Gika
- Julia Kuligowski
- Matthew Lewis
- Jonathan Mosley
- Candice Ulmer
- Dajana Vuckovic
- Ian Wilson
- Krista Zanetti

Quality Assurance

- Annie Evans
- Oliver Fiehn
- Michael Herold
- Matthew Lewis
- María Eugenia Monge
- Jonathan Mosley
- Sindhu Nair
- Oliver Schmitz
- Panteleimon Takis

The Quality of Metabolite Annotation and Identification in LC-MS-based Untargeted Metabolomics

WARWICK (RICK) DUNN

ON BEHALF OF THE mQACC FORUM SERIES ORGANIZERS

VIRTUAL INTERACTIVE FORUM HELD ON NOV 30, 2021

Metabolomics Quality Assurance & Quality Control Consortium (mQACC)

What do we mean by annotation and identification?

IDENTIFICATION matches two or more complementary types of data collected for a biological sample to data collected with chemical standards applying the same analytical methods (for example, MS/MS and RT matches to an in-house library) – higher confidence

ANNOTATION matches one or more complementary types of data collected for a biological sample but not to data collected with chemical standards applying the same analytical methods (for example, MS1 match to HMDB, MS/MS match to mzCloud) – lower confidence

We will use the term IDENTIFICATION in this workshop to include both annotation and identification

References

L. Sumner et al., Metabolomics (2007), https://link.springer.com/article/10.1007/s11306-007-0082-2

1. What type of data do you use to identify metabolites (choose all that apply)?

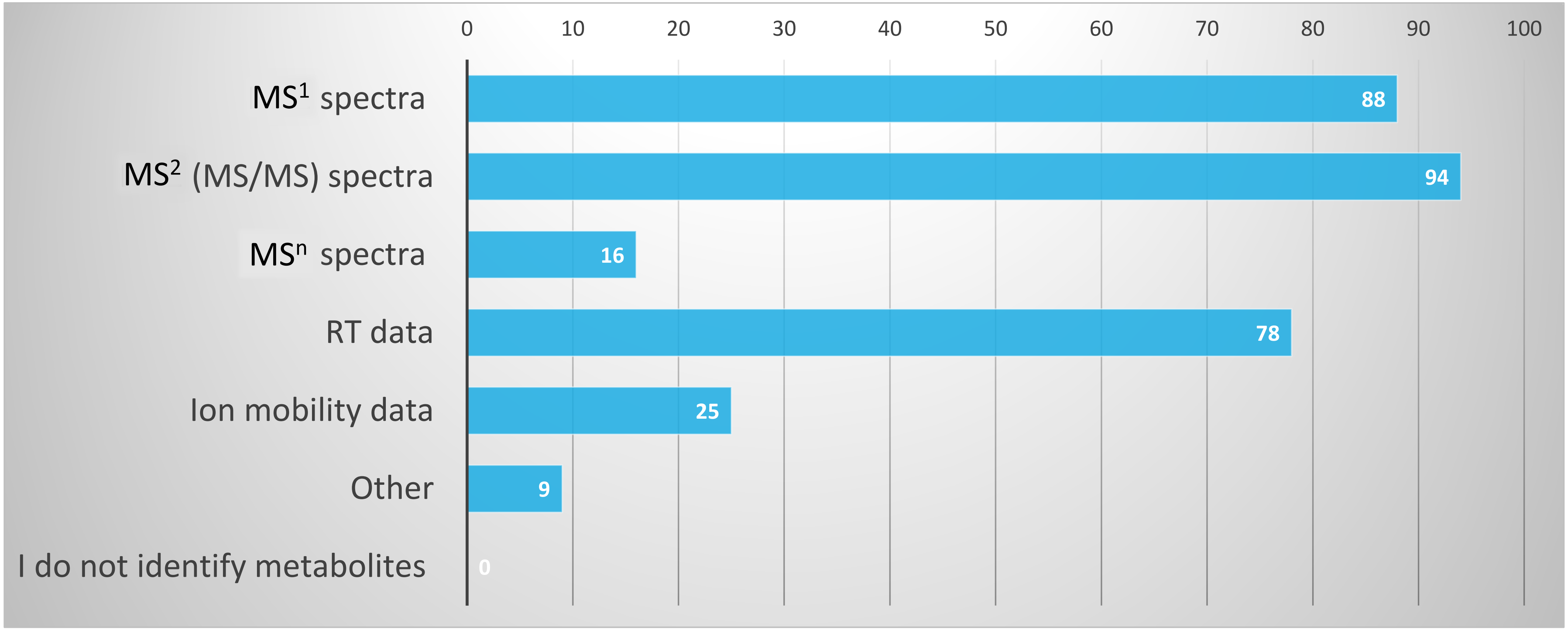

2. What mass accuracy criterion at MS1 do you use during your metabolite identification?

Metabolomics Quality Assurance & Quality Control Consortium (mQACC)

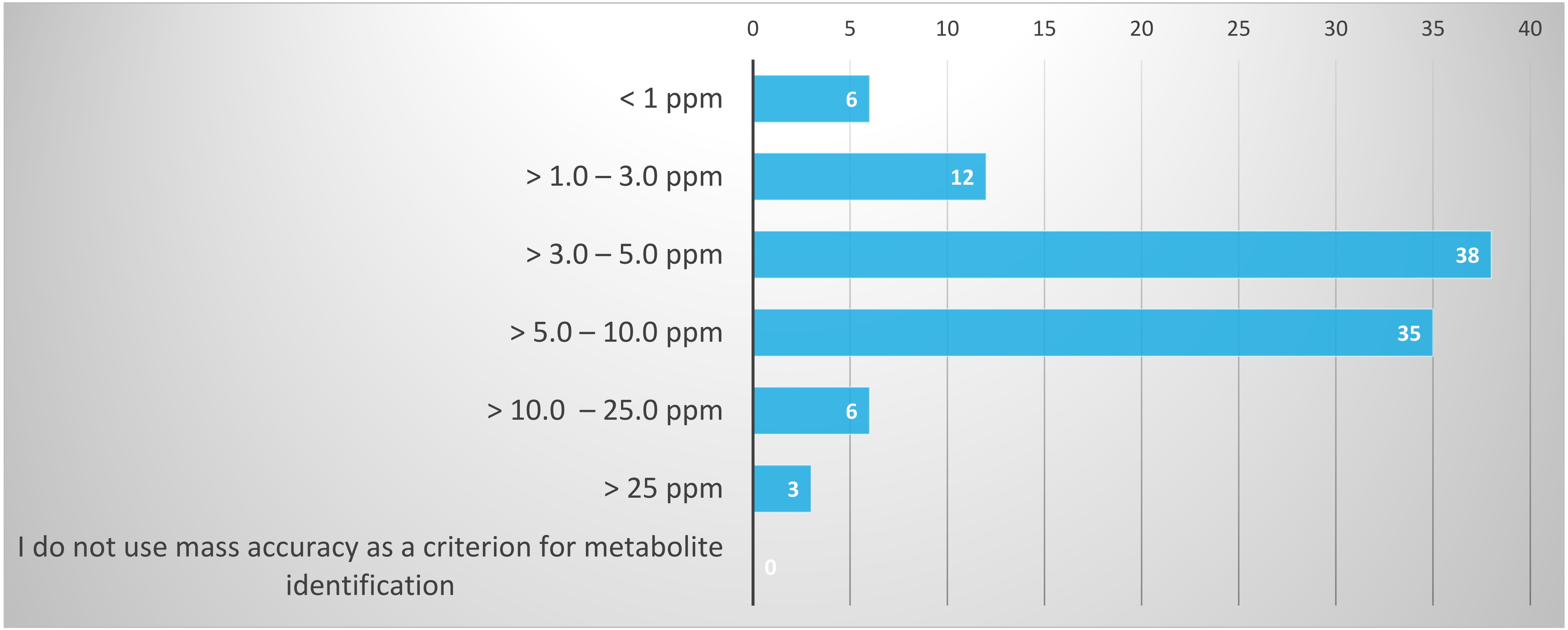

3. What type of RT and MS/MS libraries do you use to annotate/identify metabolites (choose all that apply)?

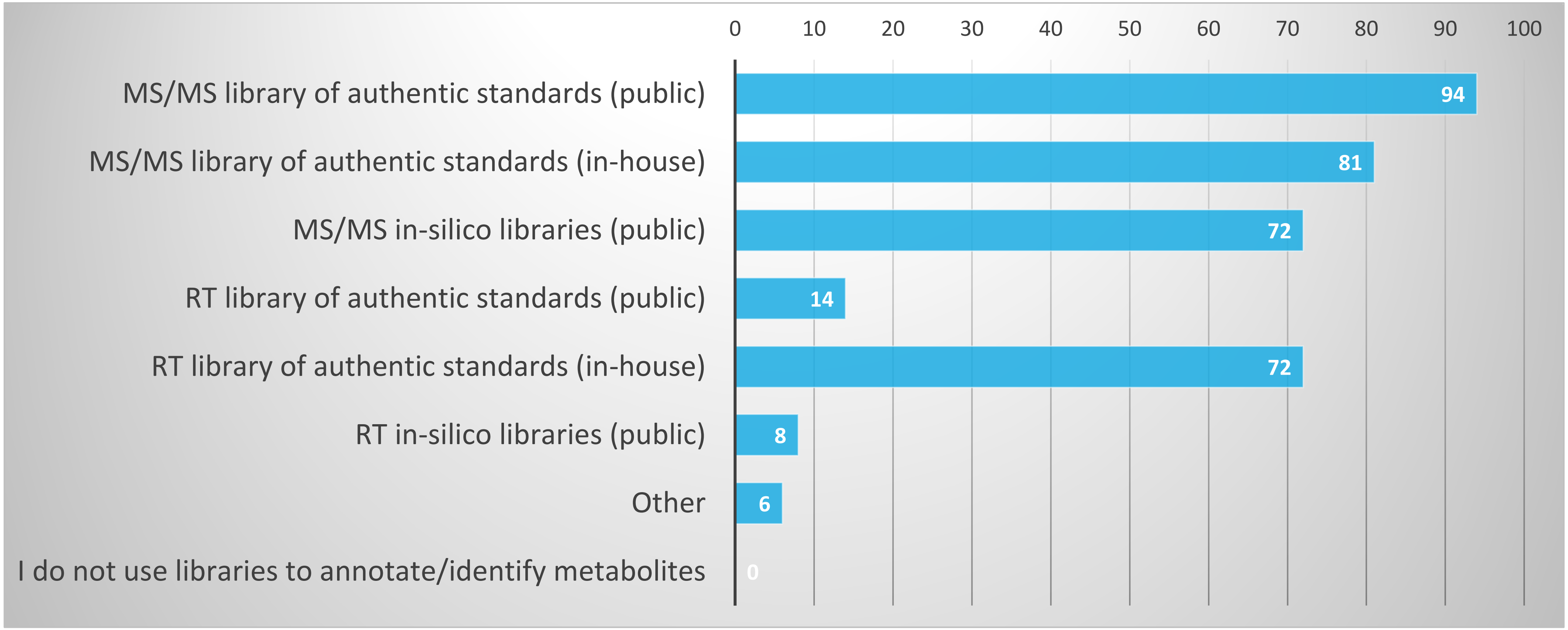

4. How many metabolites do you typically identify with in-house (MS/MS and RT) LC-MS libraries?

Metabolomics Quality Assurance & Quality Control Consortium (mQACC)

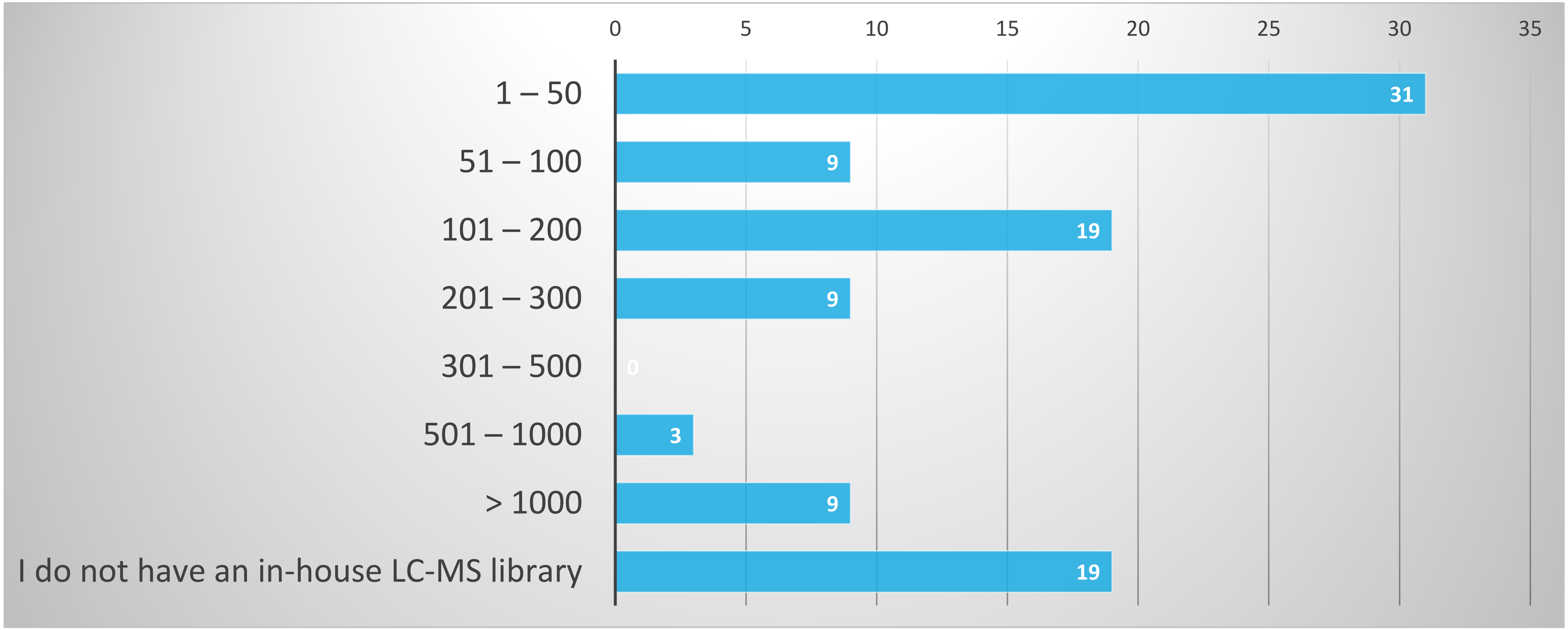

5. How do you confirm separate detection of isomers in your identification in the case of multiple isomers (choose all that apply)?

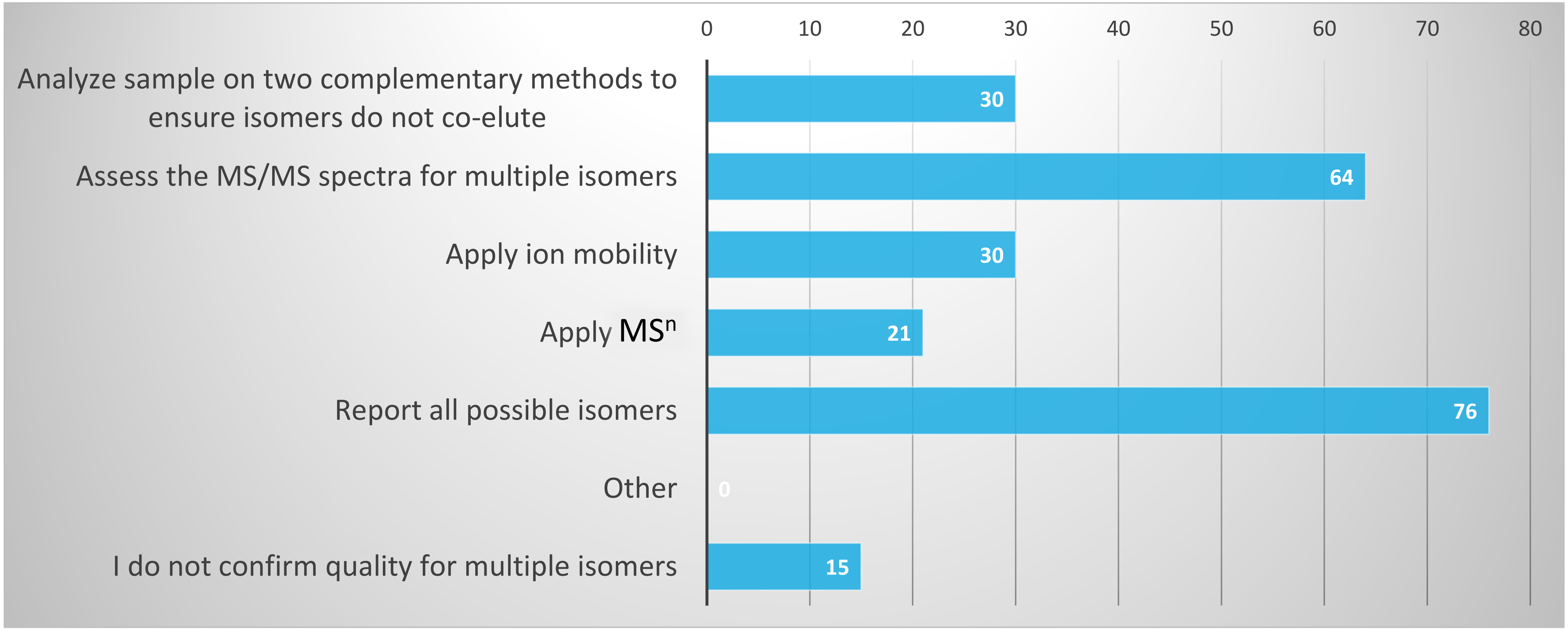

6. Which quality indicators do you use to assess your confidence in metabolite identification (choose all that apply)?

Metabolomics Quality Assurance & Quality Control Consortium (mQACC)

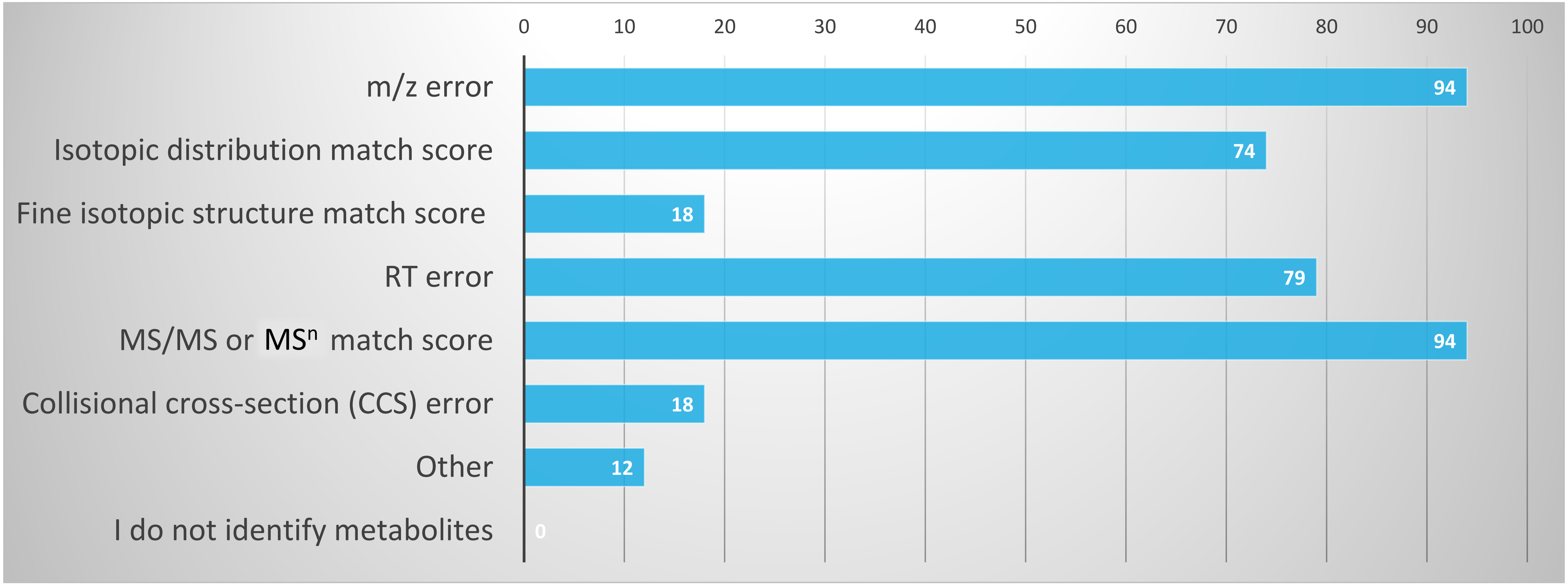

Evidence and criteria

Evidence

e.g., report RT of metabolite in biological samples and in RT library

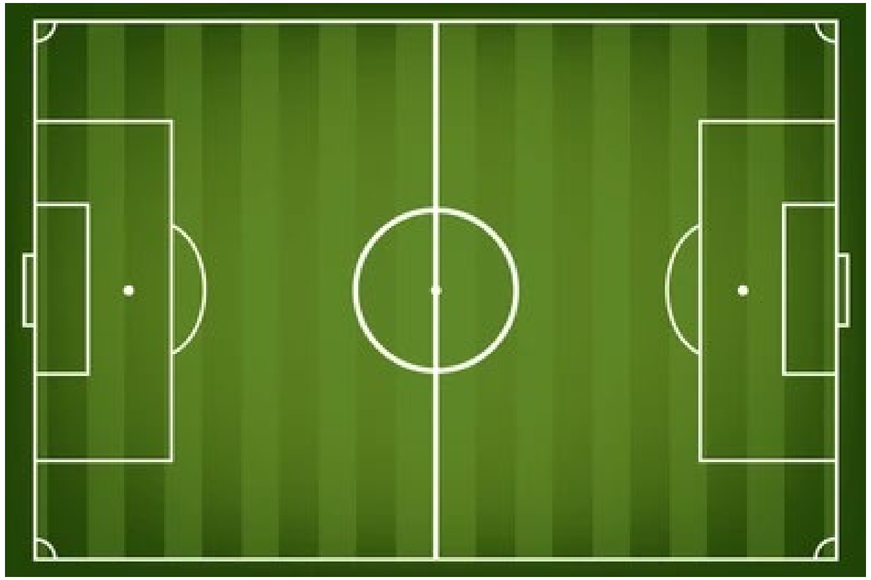

Criteria

e.g., report m/z error used for MS^1-based identification

Polling Question

Instructions for participating in Polling

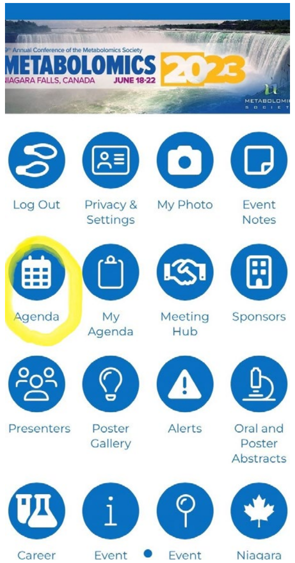

Step 1. Open up the app and select “Agenda”

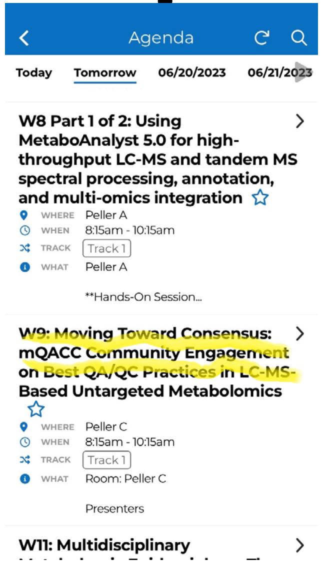

Step 2. Select “W9.” in the agenda

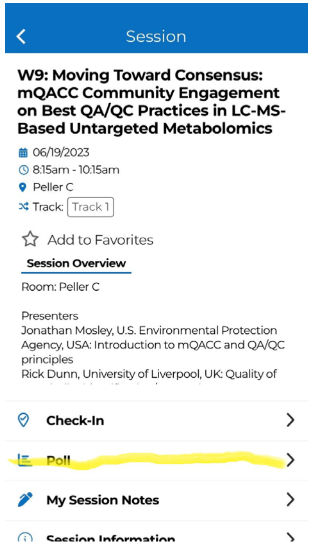

Step 3. Click the arrow next to “Poll”

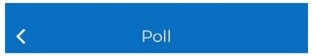

Step 4. The page will remain blank until Polling is activated by the moderator.

Q1 When reporting metabolite identifications, which of the following should be provided?

a. Quality criteria only (e.g., acceptable MS^1 mass accuracy)
b. Experimental evidence only (e.g., RT in biological sample and RT library)
c. Both quality criteria and experimental evidence
d. Neither quality criteria nor experimental evidence
e. I do not report metabolite identifications

Q2 Guidance for the quality of metabolite annotation and identification in untargeted LC-MS-based metabolomics studies in upcoming mQACC guidelines should cover (choose all that apply):

a. Considerations in choosing quality criteria
b. Types of evidence including use-case scenarios
c. Evaluation metrics
d. Reporting guidelines
e. Other
f. I do not think that guidance for this topic should be included

The Use of Reference Materials in LC-MS-based Untargeted Metabolomics

TRACEY SCHOCK

ON BEHALF OF THE mQACC FORUM SERIES ORGANIZERS

VIRTUAL INTERACTIVE FORUM HELD ON MAR 10, 2022

Metabolomics Quality Assurance & Quality Control Consortium (mQACC)

Not about general QC materials!

Reference Material (RM) – A homogeneous, stable material well-characterized for one or more chemical and/or physical properties[1].

Distinctly different from other types of QC materials, both in composition and purpose(s) for use.

Example RMs

- Certified/Standard RMs[1]
- Commercial RMs
- Long-term RMs

Not RMs

- Pooled QC (intrastudy) samples
- Process blanks
- Solvent blanks

References

[1]International Organization for Standardization. (2016). *General requirements for the competence of reference material producers* (ISO Standard No. 17034). Retrieved from https://www.iso.org/standard/29357.html

Composition and Source of RMs

How do you obtain your RM? (choose all that apply)

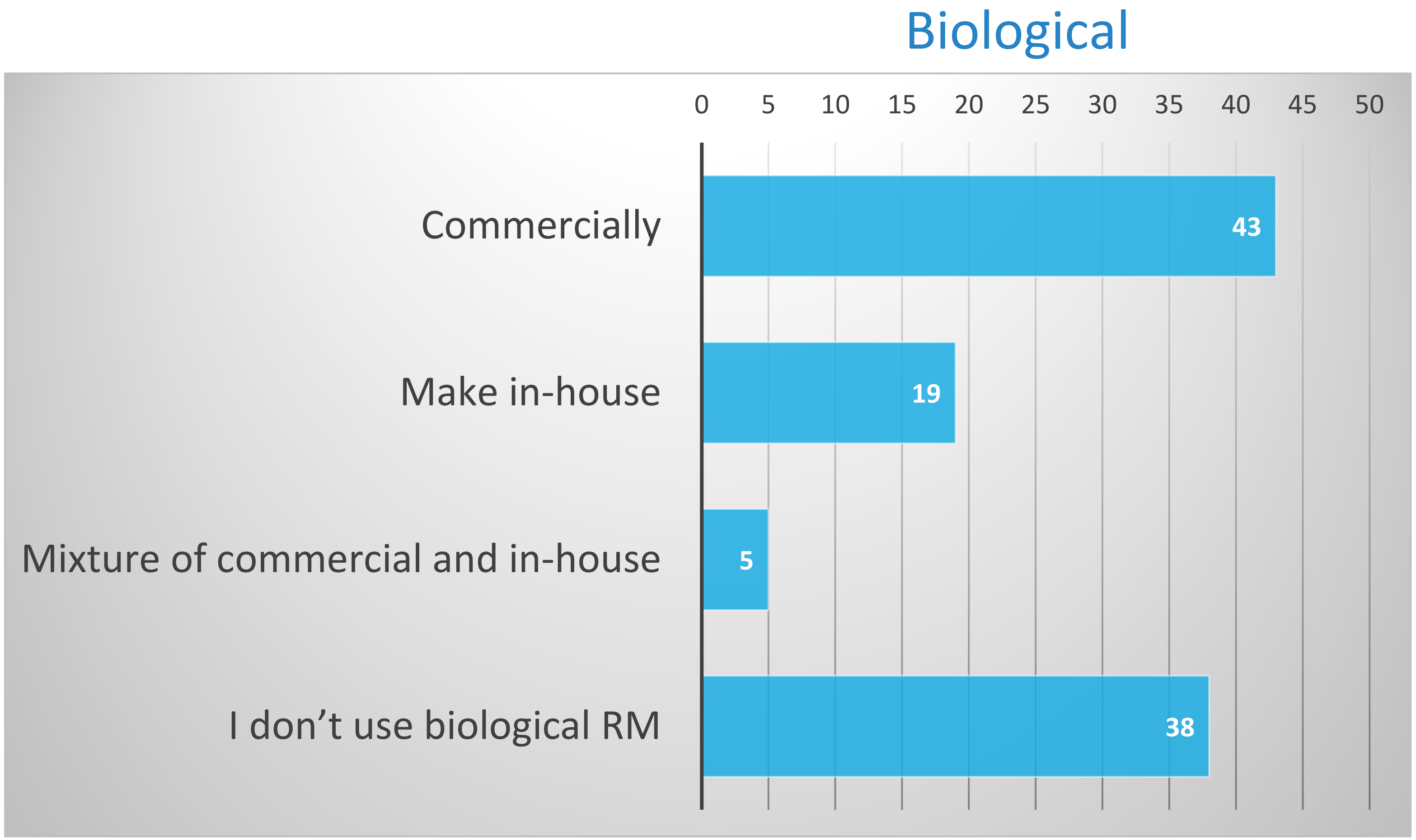

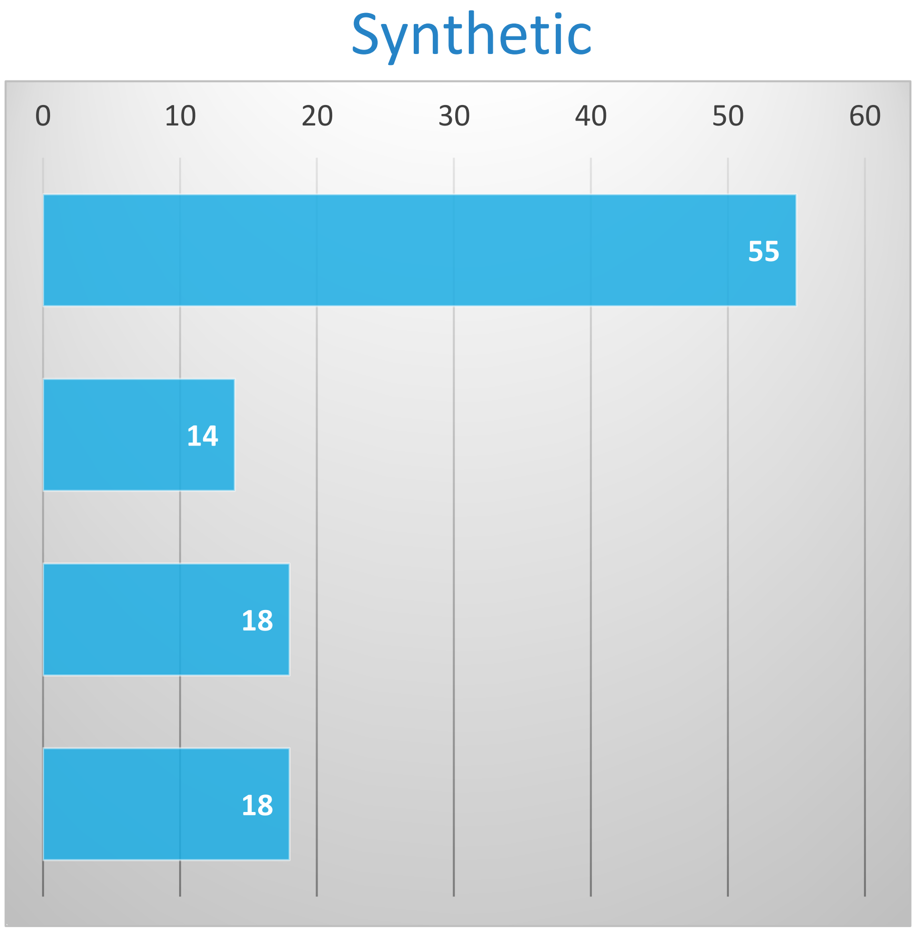

Metabolomics Quality Assurance & Quality Control Consortium (mQACC)

Follow up question:
Provide examples of Biological and Synthetic RMs used

Biological

NIST National Institute of Standards and Technology

SRM 1950

Metabolites in Frozen Human Plasma

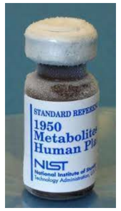

MilliporeSigma

BIOIVT ELEVATING SCIENCE®

Synthetic

Avanti® Polar Lipids
Part of Croda International Plc

Mixture of Standards (In-house)

Mixture

IROA TECHNOLOGIES
...making metabolic profiling easy®

Yeast extract +
mixture of standards

NIST SRM 1950 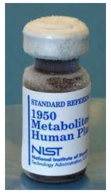 +
mixture of standards

Metabolomics Quality Assurance & Quality Control Consortium (mQACC)

Reference materials for MS-based untargeted metabolomics and lipidomics: a review by the metabolomics quality assurance and quality control consortium (mQACC)

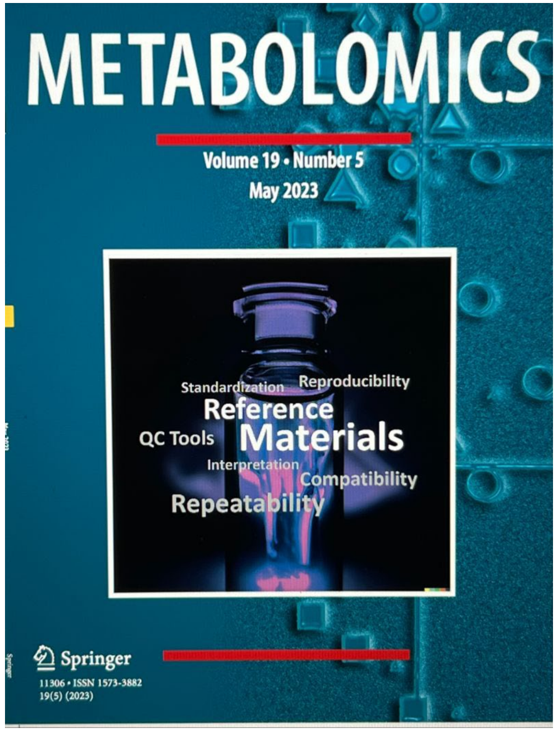

2023 Best Review Award - Metabolomics

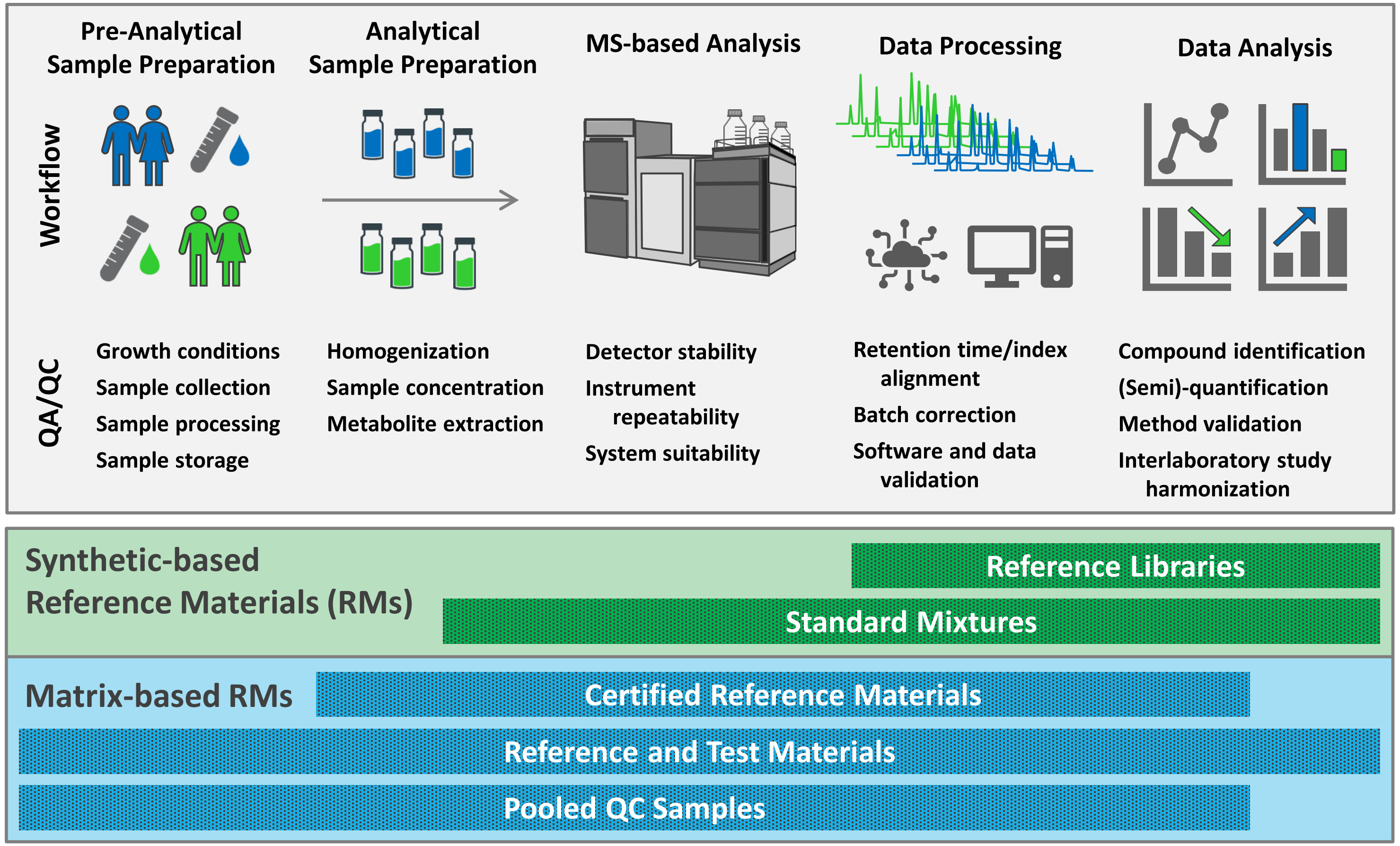

Lippa, K.A., Aristizabal-Henao, J.J., Beger, R.D. et al. Metabolomics (2022) 18:24

Important Characteristics and Purpose of RMs

Rank the most important characteristics for selecting your RM (choose all that apply).

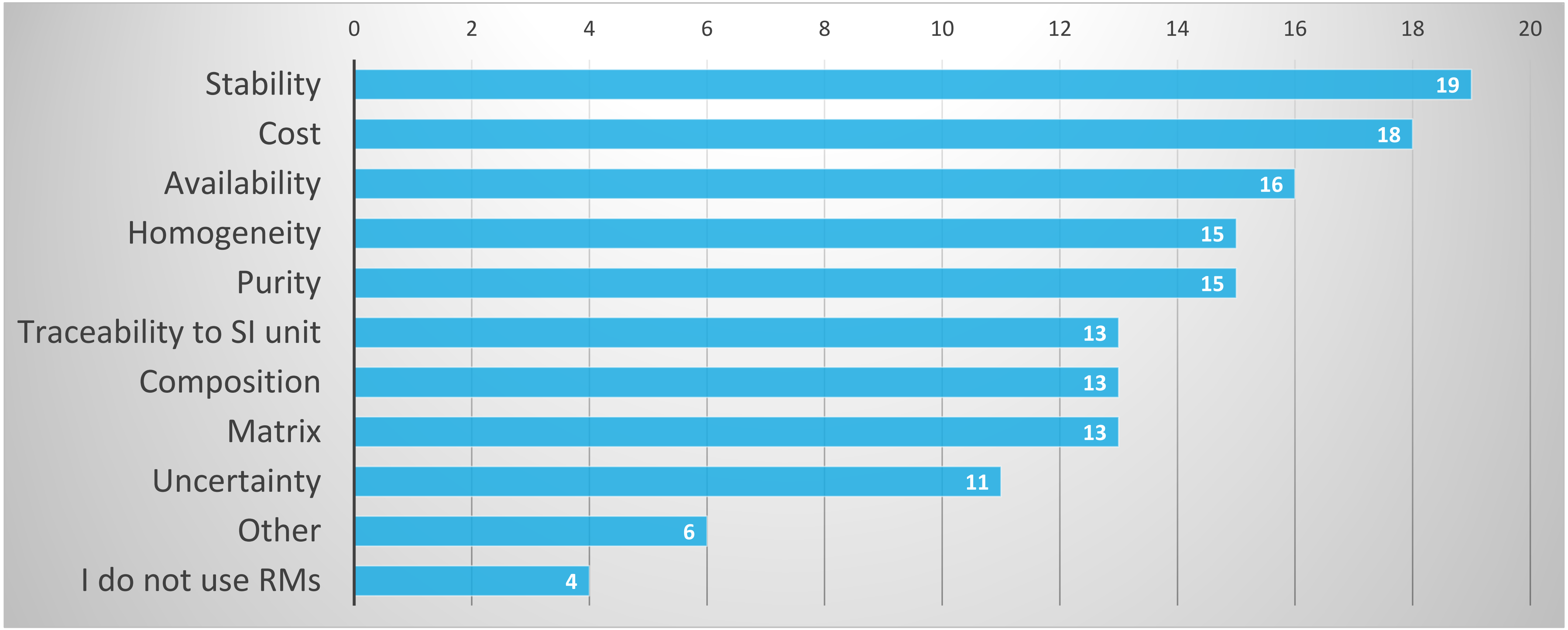

For what purpose(s) do you use RMs? (choose all that apply)

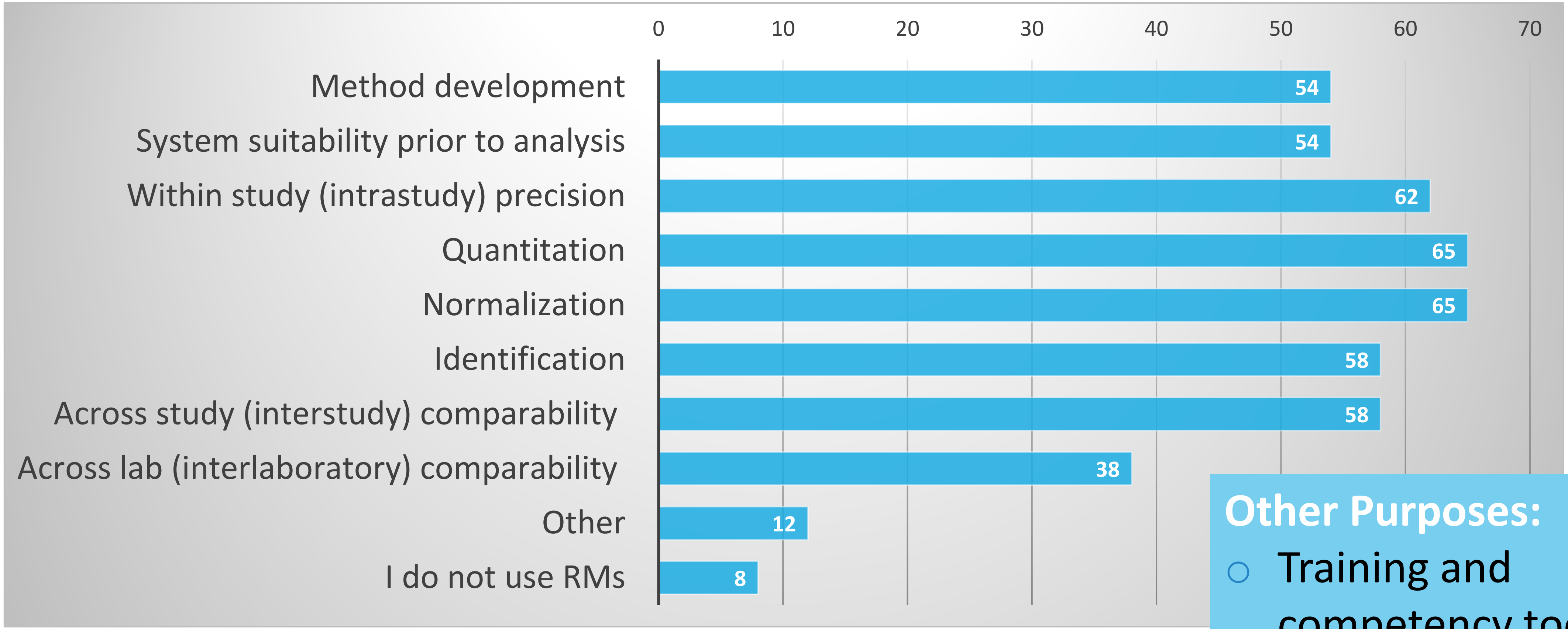

Other Purposes:

- Training and competency tool
- Retention time prediction

RM Implementation and Evaluation

Metabolomics Quality Assurance & Quality Control Consortium (mQACC)

How do you typically use your RM?

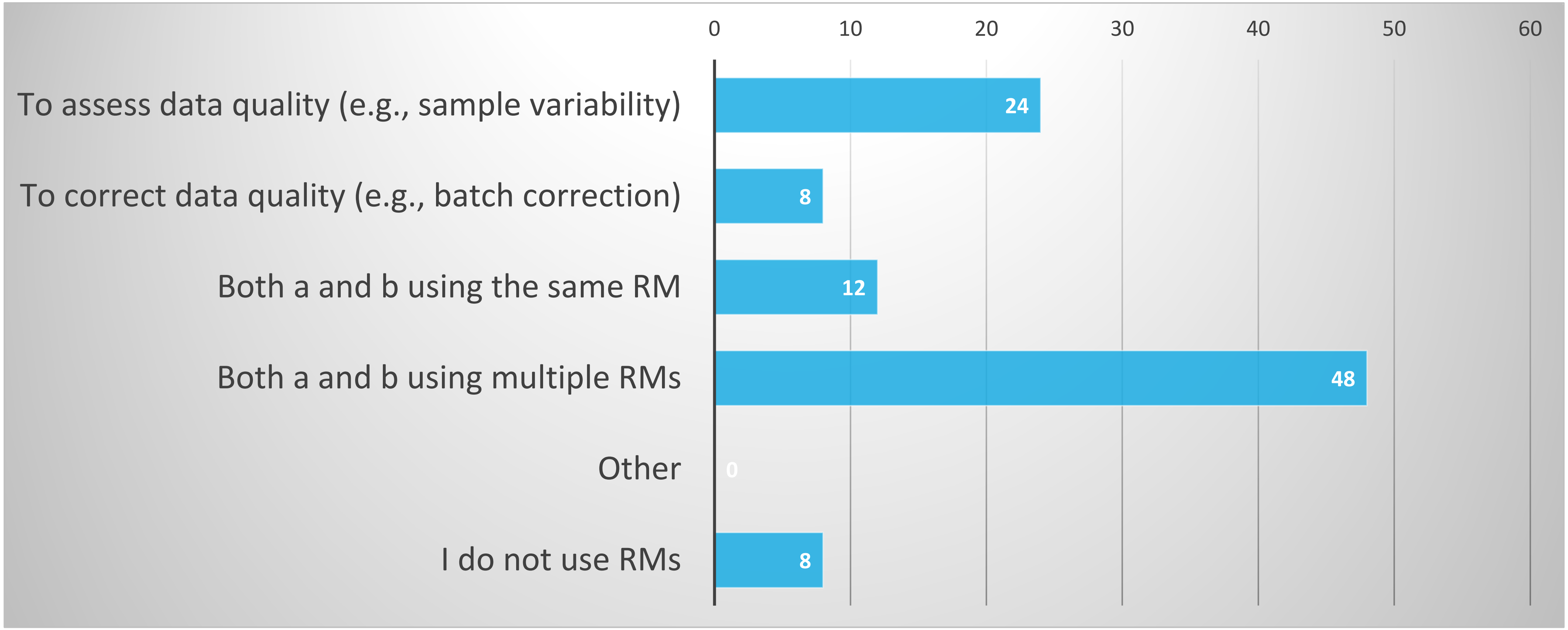

RM implementation example
Normalization

Effect of MS introduction method on Lipid Quantitation

- 2 distinct pooled plasma
- 3 introduction methods
- Same MS
- Same internal standards mix
- Analysis of 75 lipids

Normalization to a common RM

Data comparability over time and across laboratories

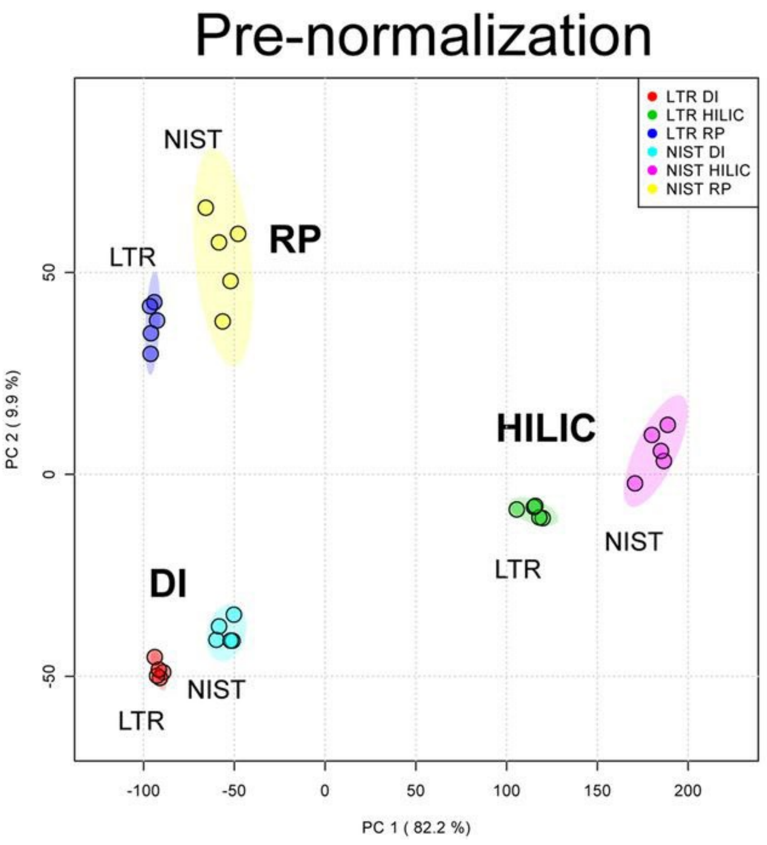

Method-dependent differences

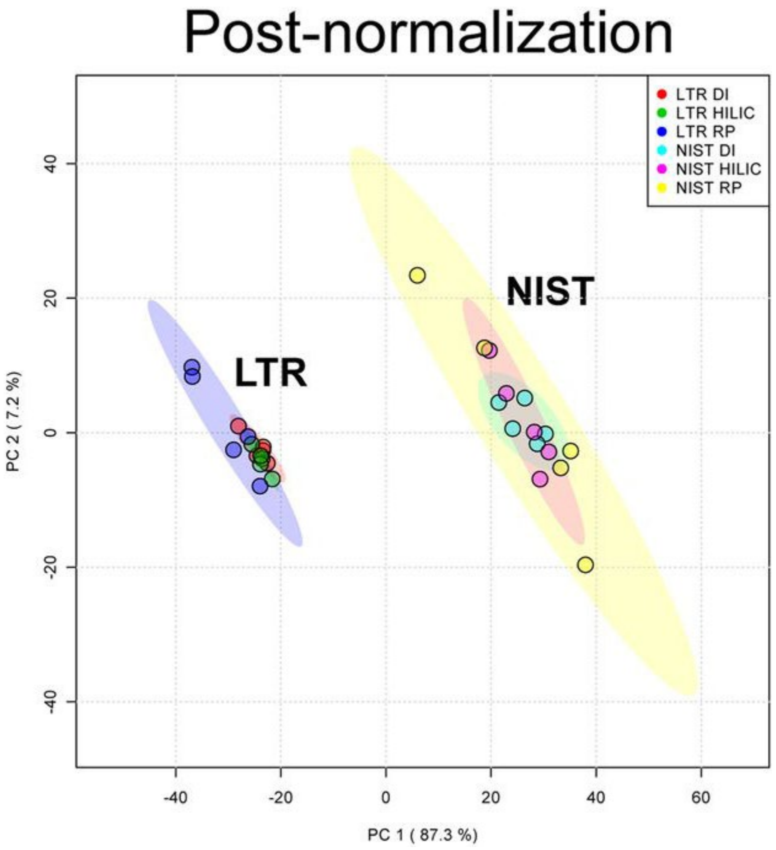

Correction for method differences

Triebl, A., B. Burla, J. Selvalatchmanan, J. Oh, S.H. Tan, M. Y. Chan, N. A. Mellet, P. J. Meikle, F. Torta, and M. R. Wenk. Shared reference materials harmonize lipidomics across MS-based detection platforms and laboratories. J. Lipid res. 2020. 61: 105–115. https://doi.org/10.1194/jlr.D119000393

What criteria do you use to compare data from your RM samples over time? (choose all that apply)

Metabolomics Quality Assurance & Quality Control Consortium (mQACC)

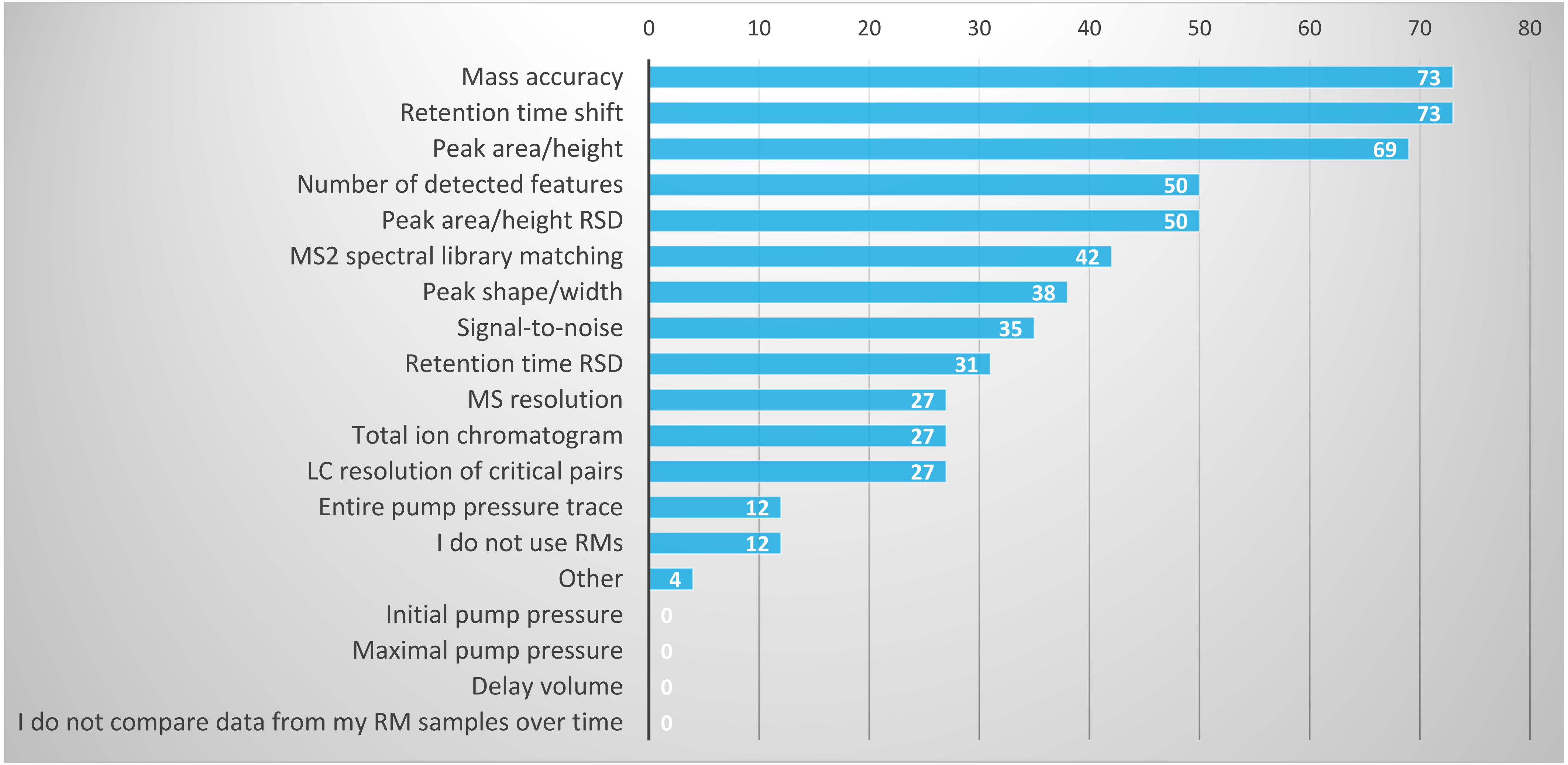

RM Injection

When do you inject your RM (NOT pooled QC) sample during an analytical batch? (choose all that apply)

Metabolomics Quality Assurance & Quality Control Consortium (mQACC)

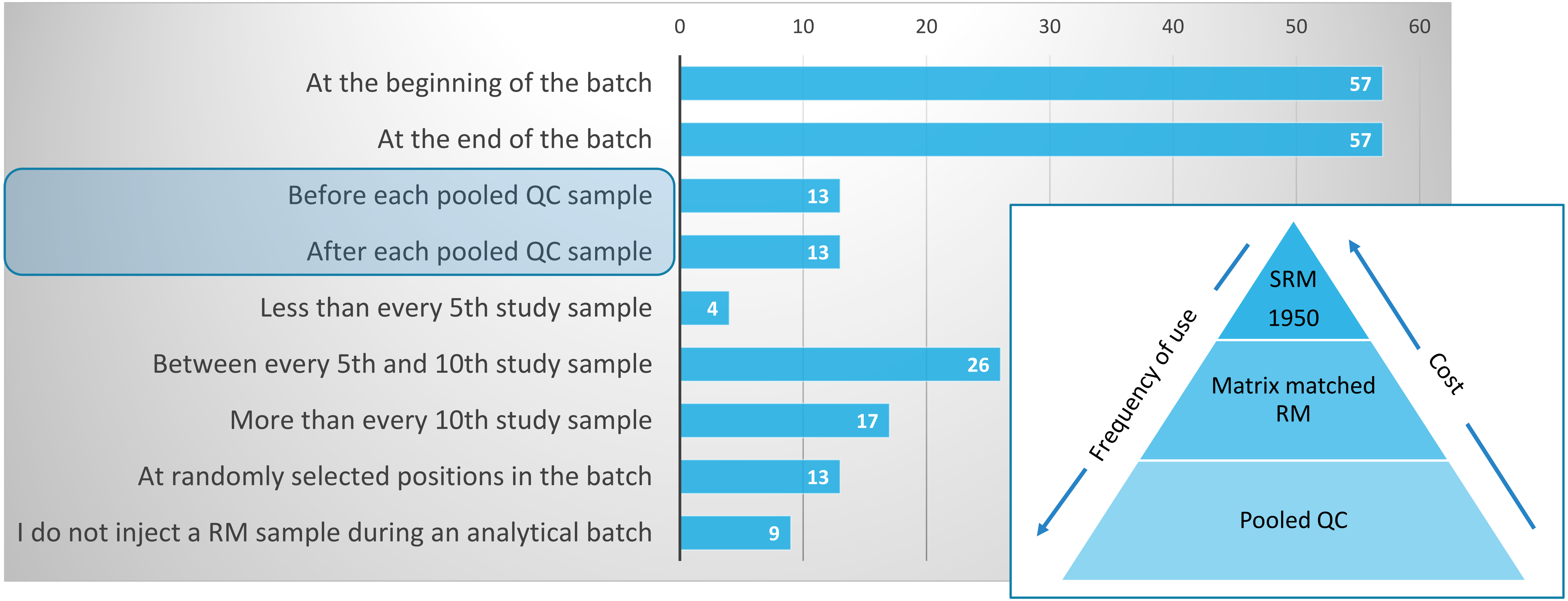

Case Study: Redundancy of QC samples

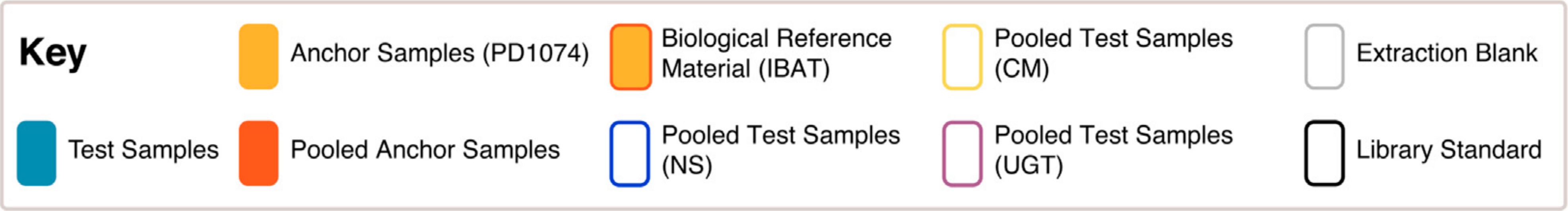

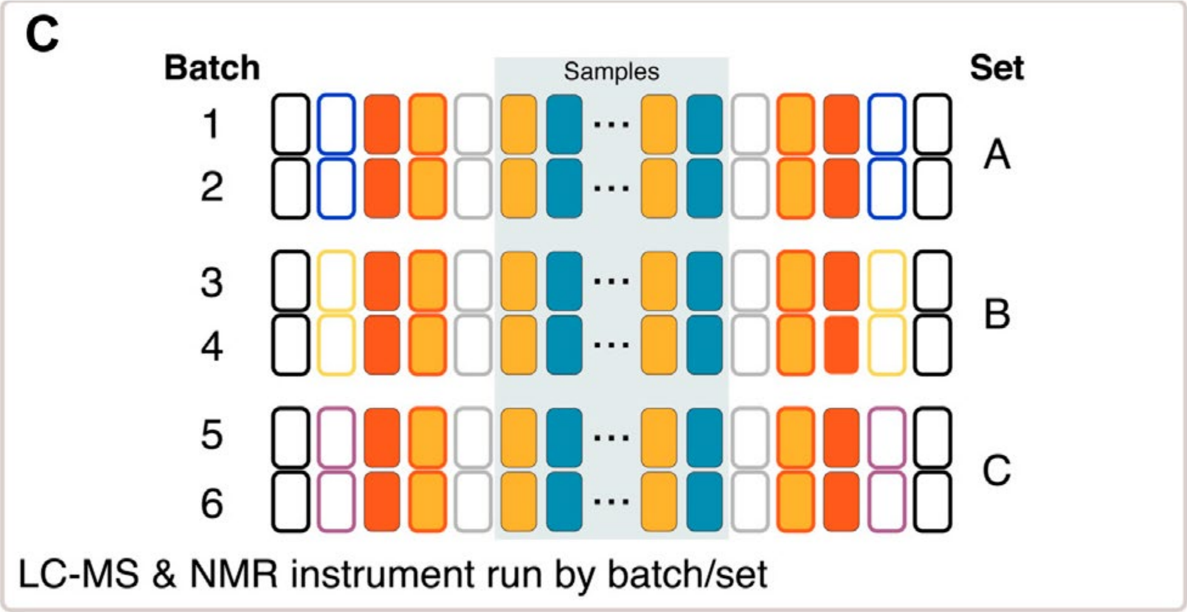

- 6-batch run
- Strict criteria: include only features detected in 100 % QCs
- Run 6 had very few features to pass
  - QC failure across all QC samples
- Used a different sample type (matrix-matched reference material) to accept their batch
  - Similar composition of two sets of samples and study samples
  - Conclude that study samples and all other quality controls were ok

Redundancy in QC strategy is important

Shaver AO, **Garcia BM**, Gouveia GJ, Morse AM, Liu Z, Asef CK, Borges RM, Leach FE, Andersen EC, Amster IJ, Fernández FM, Edison AS And Mcintyre LM (2022) An Anchored Experimental Design And Meta-analysis Approach To Address Batch Effects In Large-scale Metabolomics. *Front. Mol. Biosci.* 9:930204. DOI: 10.3389/Fmolb.2022.930204

What barriers inhibit reference materials use?

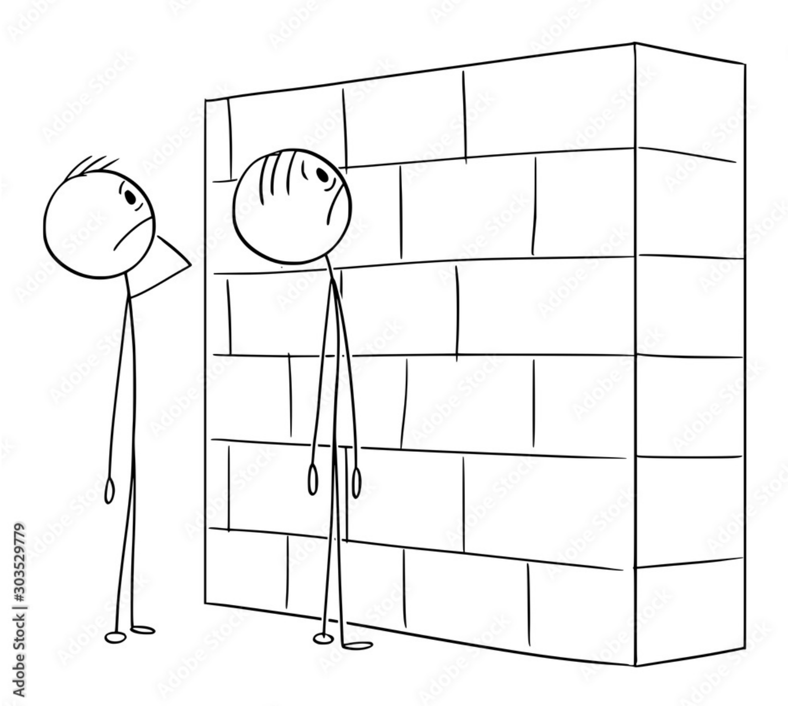

Low RM use
- Do not use RMs
  - 8-17% Forum respondents
  - 33 % mQACC survey respondents

Request for more certified materials
- Mouse organs
- More plants

Standard mixes
- Do not fit needs of all research
- Mix and match mixtures- “Clunky”
- Solubility difficulties to cover chromatographic range

↑ Certification

↑ Cost

More educational information needed

- Reference materials serve multiple purposes within a metabolomics workflow
- RM composition and characteristics define application
- Injection frequency and placement are specific to purpose
- Encourage reporting RM use, data and evaluation

Metabolomics

Quality Assurance & Quality Control

Consortium (mQACC)

Polling Question

Q3 The use of reference materials in untargeted LC-MS-based metabolomics studies in upcoming mQACC guidelines should cover (choose all that apply):

a. Considerations in choosing a reference material
b. Purpose descriptions including use-case scenarios
c. Injection frequency and placement within a run
d. Evaluation metrics
e. Reporting guidelines
f. Other
g. I do not think guidance for this topic should be included

Data Quality Review in LC-MS-based Untargeted Metabolomics

DAJANA VUCKOVIC

ON BEHALF OF THE MQACC FORUM SERIES ORGANIZERS

VIRTUAL INTERACTIVE FORUM HELD ON MAY 26, 2022

Metabolomics Quality Assurance & Quality Control Consortium (mQACC)

Important terms related to data quality review

Analytical Batch – A series of continuously analyzed samples

Internal Standards – Exogenous or labelled endogenous compounds which are intentionally spiked into some or all samples in a process and are used to help assess data quality.

Quality Control (QC) Sample – Any of a various type of mixtures prepared to assess the quality of untargeted data. QC sample is a generic term.

Example QC samples

- Pooled (intrastudy) QC (**95%**)
- Phenotypic QC (**35%**)
- Reference material (e.g., NIST 1950) (**75%**)
- Mixtures of standards (**80%**)

Also includes blanks!

- Process blank (**75%**)
- Solvent (true) blank (**85%**)
- Other (e.g., lyophilization)

References

D. Broadhurst et al., Metabolomics (2018)
https://doi.org/10.1007/s11306-018-1367-3

Evans, A.M., et al. Metabolomics (2020)
https://doi.org/10.1007/s11306-020-01728-5

Real-time versus post-acquisition data review

Real-time Data Review

- Is batch running well?
- Are there any issues that can be addressed during the run?
- Should the run be stopped?
- Which criteria will trigger an immediate action?

Early detection of problems usually using selected metabolites or internal standards

Post-acquisition Data Review

- Is the batch quality acceptable for further biological and statistical interpretation?
- Which criteria will be used to accept or reject a batch?
- Which criteria will be used to detect sample outliers?

Holistic data review of all features/metabolites and sample types

Which of these do you use to assess data quality in quality control samples (choose all that apply)

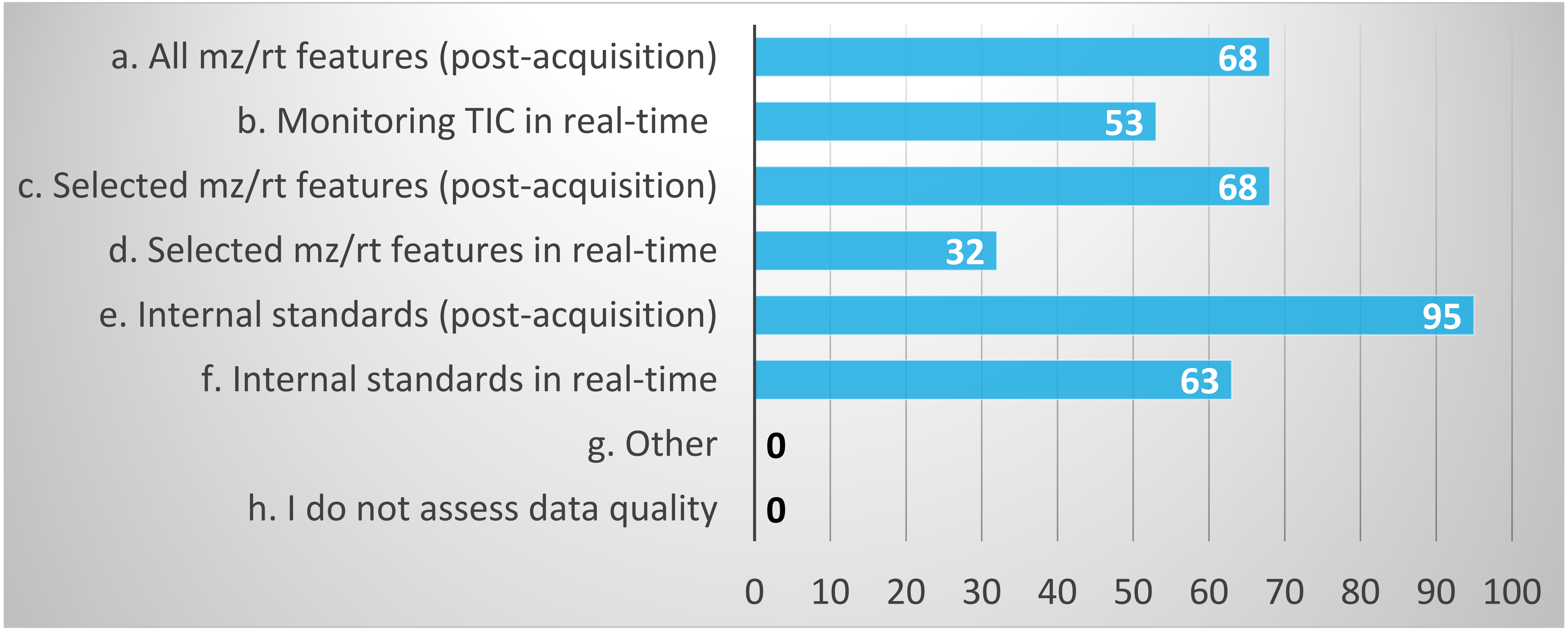

I assess the following data quality metrics in QC samples (choose all that apply):

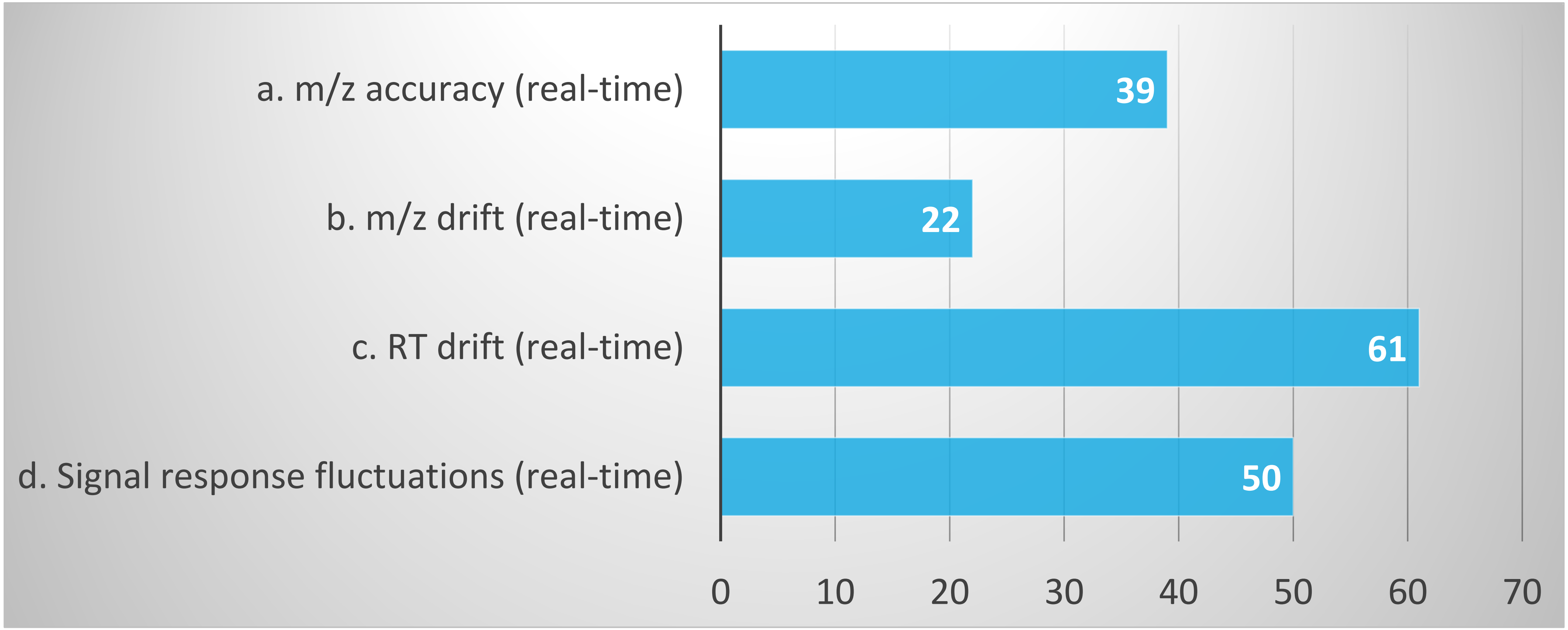

(cont'd) I assess the following data quality metrics in QC samples (choose all that apply):

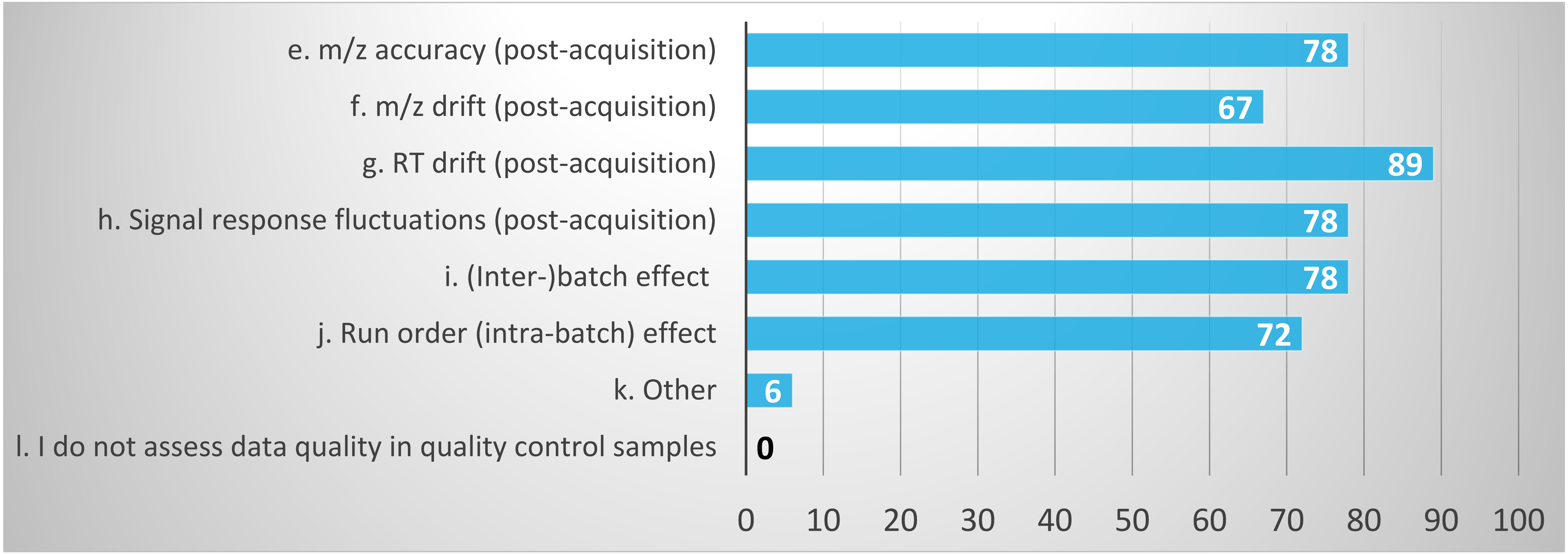

What are the typical acceptance criteria that you apply (choose all that apply)?

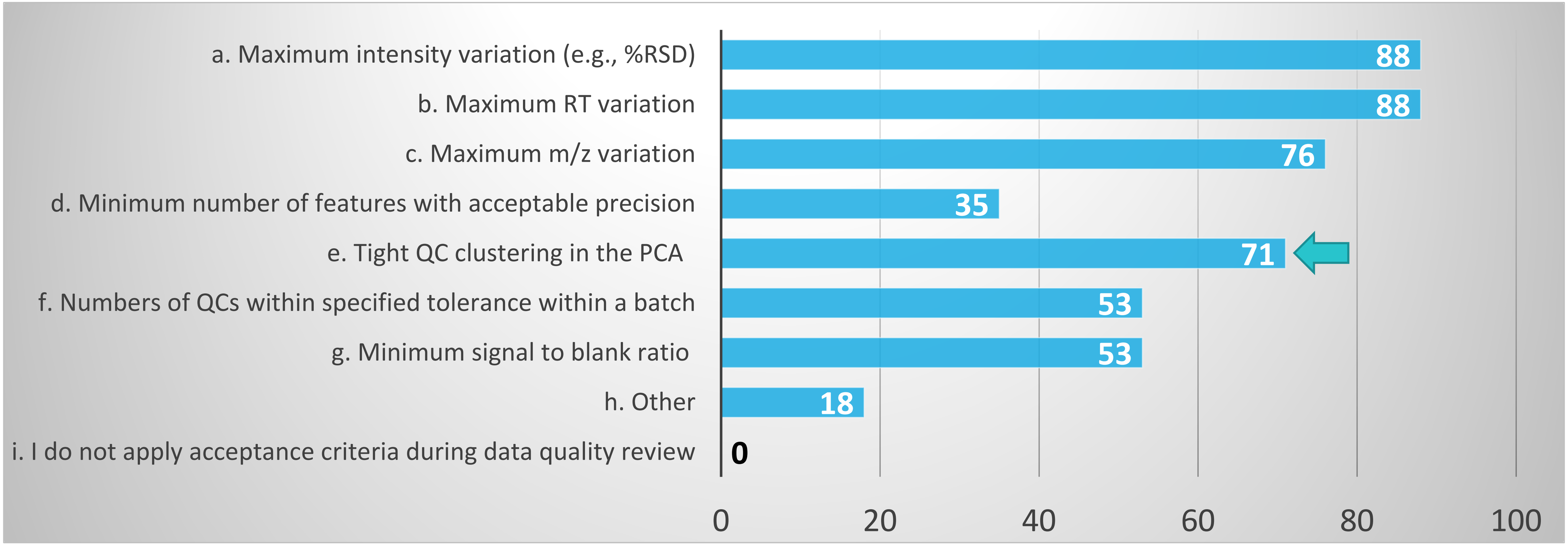

Batch failure and sample outlier analysis

Batch failure

- Close clustering of QCs in PCA
- Determine number of QCs within acceptance criteria

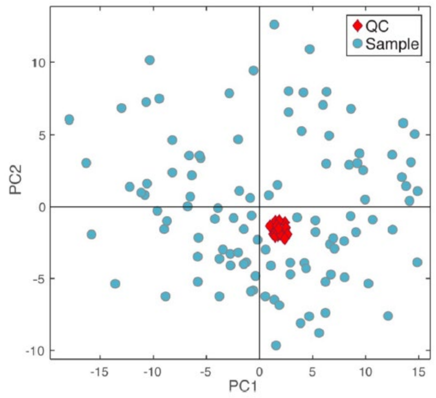

D. Broadhurst et al., Metabolomics (2018) https://doi.org/10.1007/s11306-018-1367-3

Sample outliers

- PCA (e.g. Hotelling's ellipse)
- Internal standard acceptance criteria
- Statistical outlier tests

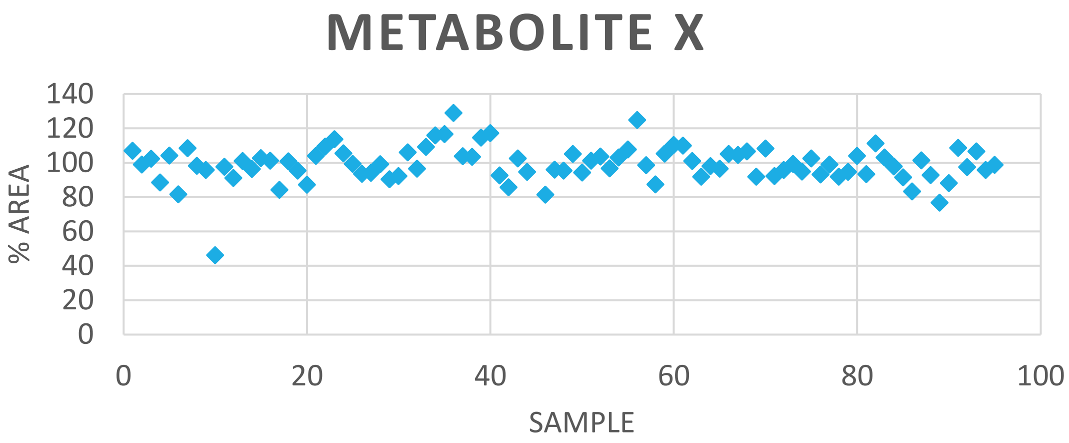

Follow-up: How do you determine number of QCs within specified tolerance? Do you use control charts or other approaches?

Metabolomics Quality Assurance & Quality Control Consortium (mQACC)

Answer 1: <u>Multi-step iterative</u> approach

- PCA
- Number of metabolites/features detected
- CV/intensity drift metrics

$$D\text{-}ratio_i = \frac{s_{i,qc}}{s_{i,sample}} \times 100\%$$

D. Broadhurst et al., Metabolomics (2018)
https://doi.org/10.1007/s11306-018-1367-3

Answer 2: Statistical metrics to assess QC

- <u>Median RSD and spread</u>
- Wary of using only PCA clustering
- D ratios - sometimes find that a batch may have good intra-day performance but inconsistent performance with other batches from the study
- D ratios - provide a quality metric that can be reported for each detected metabolite

Answer 3: combination approach

- PCA clustering + CV + standard Euclidean distance for <u>QC within and across batches</u> for inter-batch studies

Other approaches to help during failure analysis

In addition to checking clustering of QC daily, additional approaches used by a lab include:

- analyze system suitability sample in real-time to check m/z and retention time for a set of standards in SST
- One internal standard added during protein precipitation to check extraction success
- One internal standard added just prior to LC-MS analysis to monitor for injection problems
- Check time-related drift with pooled QC

Key ideas from discussion: Importance of redundancy in quality control strategies and fit-for-purpose use of specific quality control sample types

What do you do when QC samples fail acceptance criteria in an analytical batch?

Metabolomics Quality Assurance & Quality Control Consortium (mQACC)

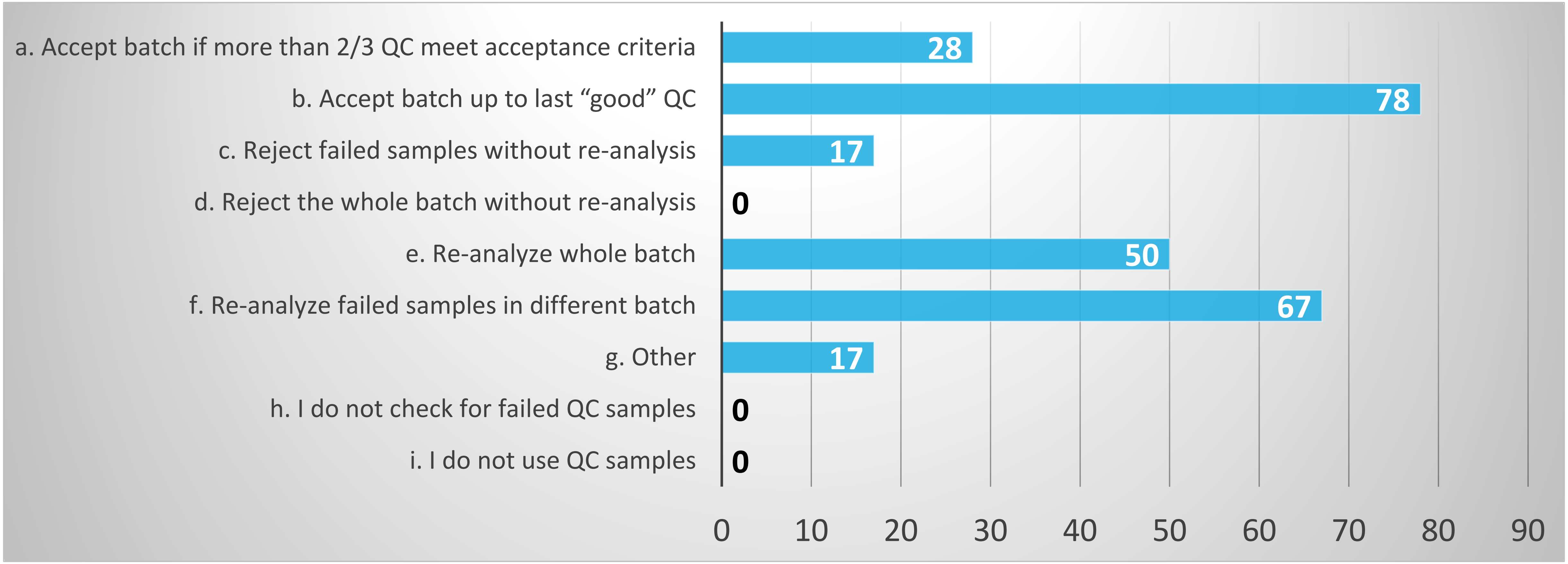

How do you detect sample outliers in your data during data quality review (choose all that apply)?

Metabolomics Quality Assurance & Quality Control Consortium (mQACC)

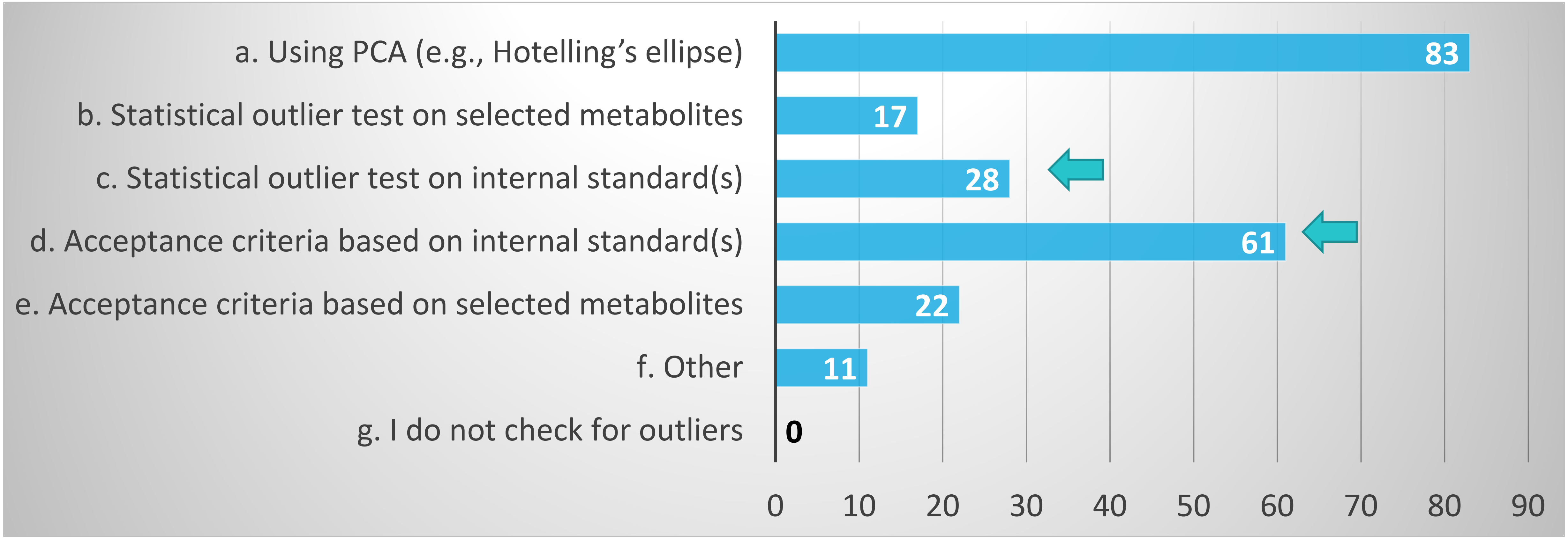

What data manipulation steps do you take to improve data quality (choose all that apply)?

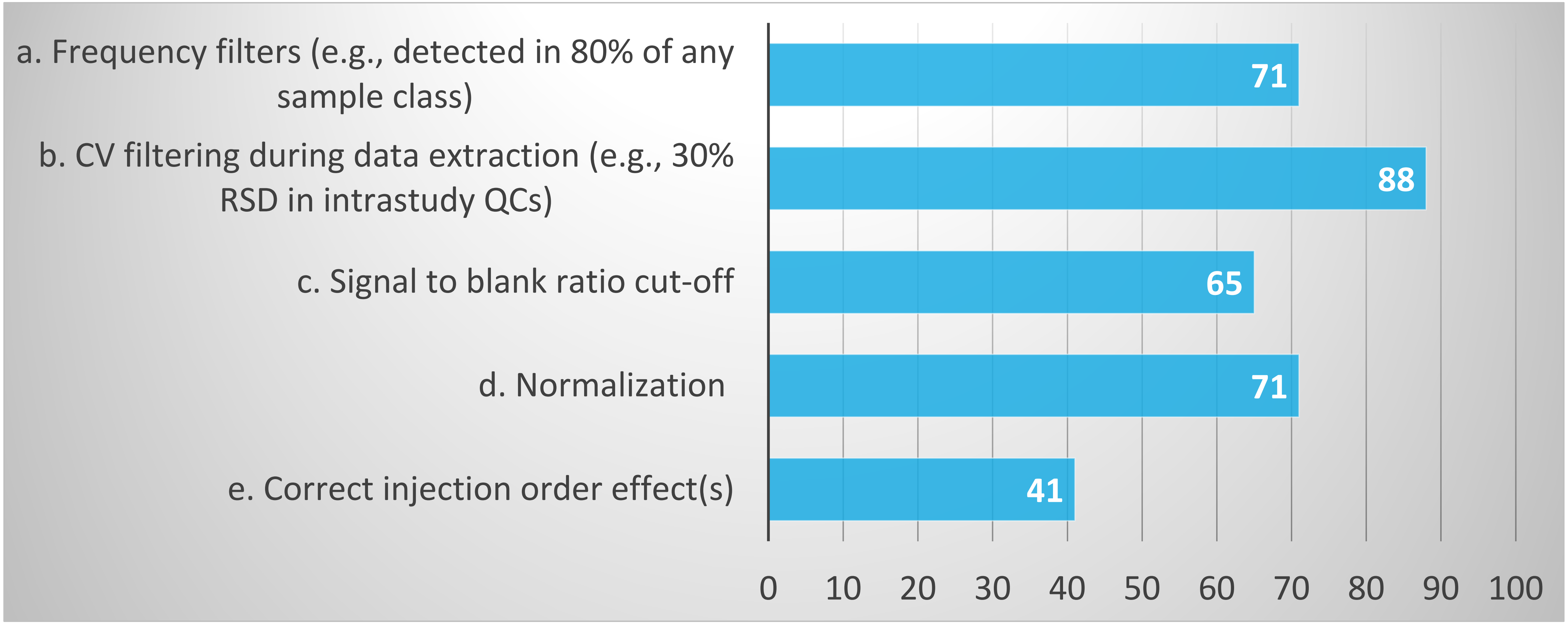

(cont'd) What data manipulation steps do you take to improve data quality (choose all that apply)?

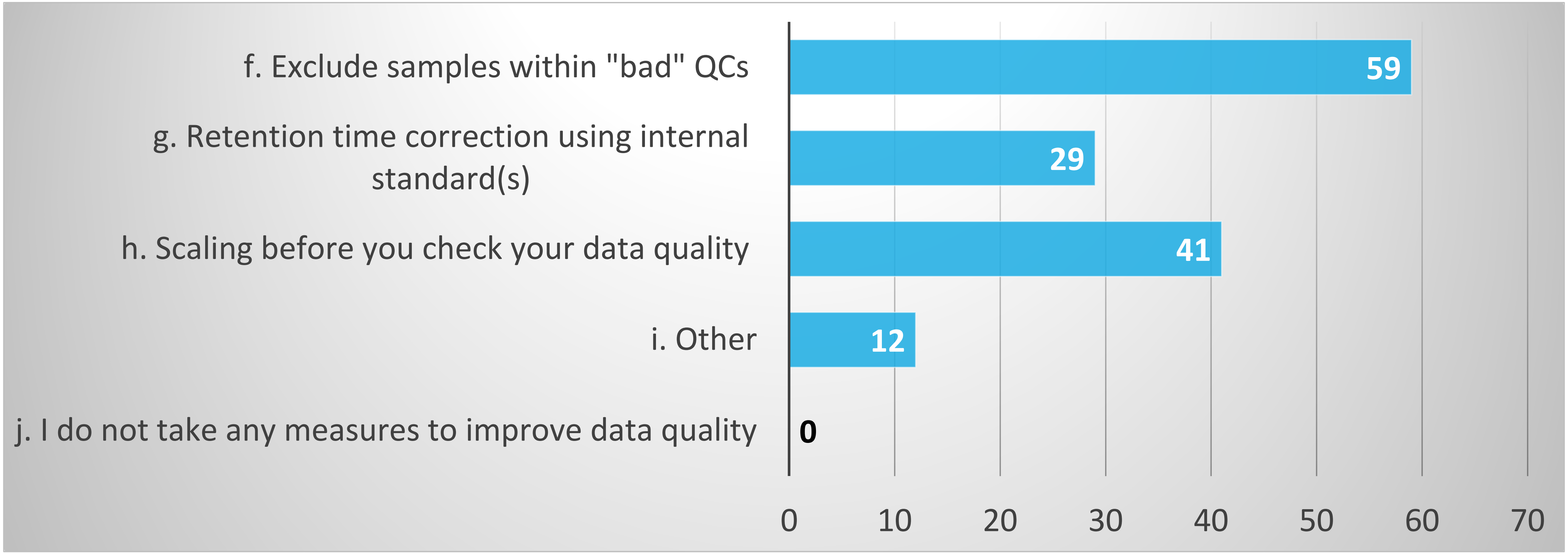

Follow-up: What decision points drive these steps?

- Do you always use the same approach or do you adapt depending on the specific study?

Adapt based on study/question/situation

Example 1:

- Not a lot of information on C. elegans metabolome so they used more stringent criteria than usually, eg, 100% frequency filter for feature selection

Example 2:

- CV depends on intensity
- 30% CV target suggested as useful guide but it is maximum not a target
- Usually markers and most useful features have much better CV

QC processes need iteration and refinement over time

Real-time monitoring

- Historical batch failures can be used to develop real-time monitoring practices and decision criteria when to stop the run

Change to QC practices in response to failure to avoid future occurrences

- In general, many respondents adopted and refined their QC practices over time
- Many practitioners assess the cause of failure and whether the failure is random or systematic
- Different types of QCs can assist failure analysis

Use of redundant practices can help minimize batch failure

- Fit-for-purpose use of various QC sample types

Polling Question

Q4 Guidance for data quality review in untargeted LC-MS-based metabolomics studies in upcoming mQACC guidelines should cover (choose all that apply):

a. Considerations in determining acceptance criteria
b. Batch failure/outlier analysis including use-case scenarios
c. Metrics and reporting guidelines for data quality review
d. Data manipulation strategies to improve data quality (e.g., feature filtering, normalization)
e. Other
f. I do not think that guidance for this topic should be included

Quality Assurance (QA)
in Untargeted Metabolomics Research

MATTHEW R. LEWIS

Metabolomics Quality Assurance & Quality Control Consortium (mQACC)

Metabolomics Quality Assurance & Quality Control Consortium (mQACC)

Quality Assurance

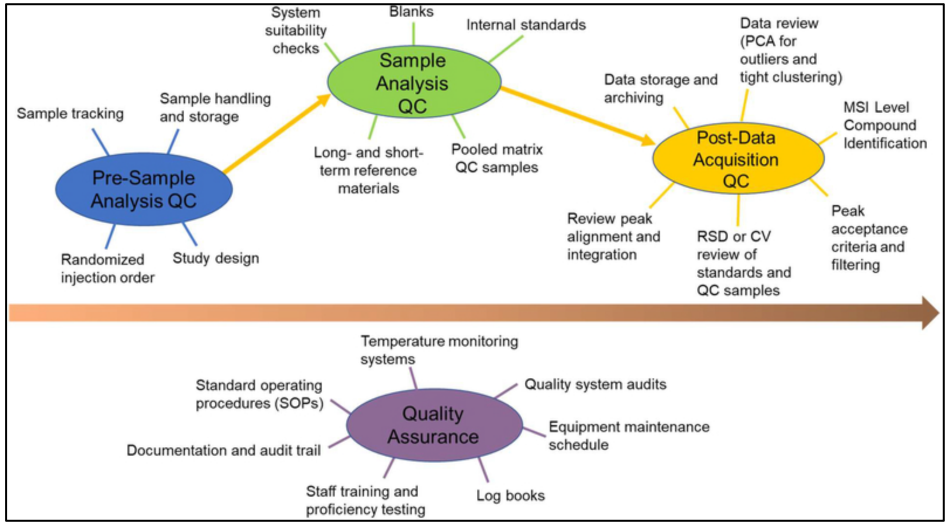

Evans, A. M., O'Donovan, C., Playdon, M., Beecher, C., Beger, R. D., Bowden, J. A., Broadhurst, D., Clish, C. B., Dasari, S., Dunn, W. B., Griffin, J. L., Hartung, T., Hsu, P. C., Huan, T., Jans, J., Jones, C. M., Kachman, M., Kleensang, A., Lewis, M. R., Monge, M. E., … Metabolomics Quality Assurance, Quality Control Consortium (mQACC) (2020). Dissemination and analysis of the quality assurance (QA) and quality control (QC) practices of LC-MS based untargeted metabolomics practitioners. *Metabolomics : Official journal of the Metabolomic Society*, *16*(10), 113. https://doi.org/10.1007/s11306-020-01728-5

QA topic	Example considerations
**Quality system audits**	• Is your quality system & QA compliance subject to routine or surprise review and inspection?
**Documentation and audit trail**	• Does a second person check data in detail?<br>• Are records formalised, organised and searchable?
**Proficiency testing**	• Are staff skills and knowledge routinely tested to ensure performance, QA system compliance and understanding?<br>• Does your lab participate in inter-laboratory studies/ring-trials?
**Standard operating procedures (SOPs)**	• Are all lab procedures well documented and controlled?
**Temperature monitoring systems**	• Is lab temperature recorded (e.g. freezer, ambient, or instrument recording systems) and archived alongside analytical data, on its own, or not at all?<br>• Is this information archived or actively used to investigate analytical phenomena (e.g. m/z or retention time drift)?
**Equipment maintenance**	• How frequently is lab equipment routinely calibrated? (weekly, monthly, between studies/users?)<br>• How is lab equipment tested and monitored? (By a designated person, by individual users?)
**Log books**	• Are the recording of daily data and observations enforced?
**Staff training**	• Are staff training records maintained (onboarding, continuous development)?<br>• Are procedures and materials in place to onboard and train personnel in untargeted metabolomics?

Quality Assurance

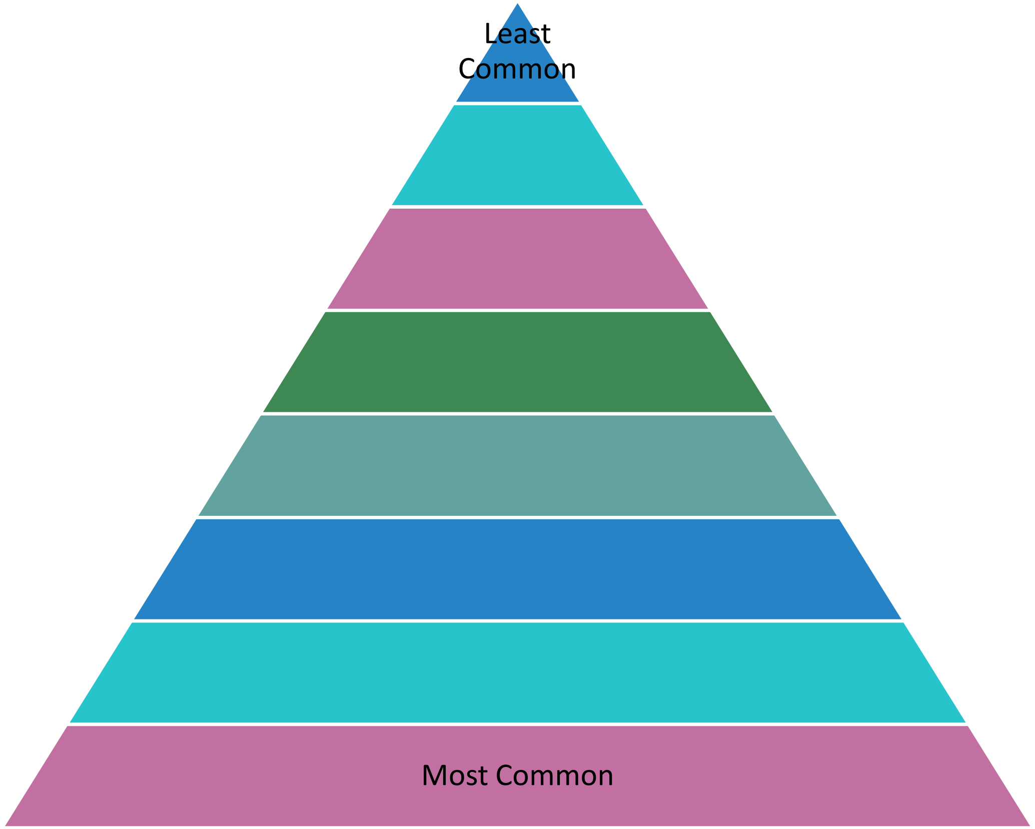

QA topic	Example considerations
**Quality system audits**	• Is your quality system & QA compliance subject to routine or surprise review and inspection?
**Documentation and audit trail**	• Does a second person check data in detail?<br>• Are records formalised, organised and searchable?
**Proficiency testing**	• Are staff skills and knowledge routinely tested to ensure performance, QA system compliance and understanding?<br>• Does your lab participate in inter-laboratory studies/ring-trials?
**Standard operating procedures (SOPs)**	• Are all lab procedures well documented and controlled?
**Temperature monitoring systems**	• Is lab temperature recorded (e.g. freezer, ambient, or instrument recording systems) and archived alongside analytical data, on its own, or not at all?<br>• Is this information archived or actively used to investigate analytical phenomena (e.g. m/z or retention time drift)?
**Equipment maintenance**	• How frequently is lab equipment routinely calibrated? (weekly, monthly, between studies/users?)<br>• How is lab equipment tested and monitored? (By a designated person, by individual users?)
**Log books**	• Are the recording of daily data and observations enforced?
**Staff training**	• Are staff training records maintained (onboarding, continuous development)?<br>• Are procedures and materials in place to onboard and train personnel in untargeted metabolomics?

Metabolomics Quality Assurance & Quality Control Consortium (mQACC)

Quality Assurance

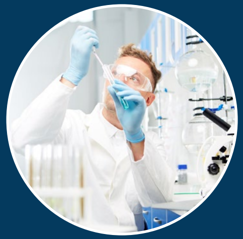

Clinical & academic research

e.g. basic, mechanistic, applied and translational research

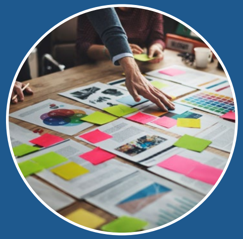

Core facilities

e.g. high volume analysis, natural products chemistry & collaborative work with academic research groups

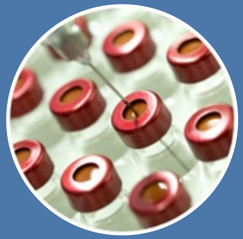

CROs & Centres

e.g. high volume analysis, multidisciplinary collaborations & services

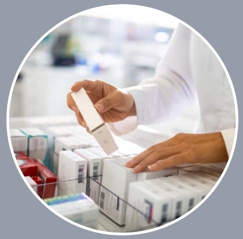

Highly regulated laboratories (e.g. Pharma, Government)

e.g. drug discovery, pre/clinical dev, drug metabolism and pharmacokinetics (DMPK), safety/efficacy, metID

Quality Assurance & Quality Control

What is fit for purpose?

Bioanalytical Mass Spectrometry Lab
Center for Bionanoscience Research (CIBION)

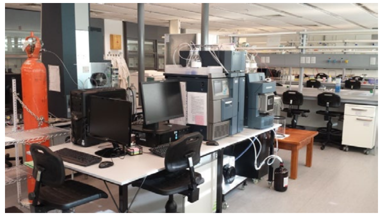
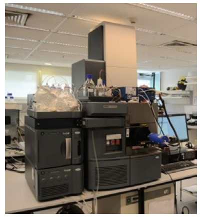
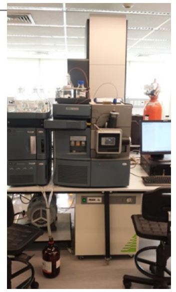

Dr. María Eugenia Monge

PI Bioanalytical Mass Spectrometry Group
Research staff CONICET at CIBION

CONICET
CIBION

Topics of focus:

1. Documentation and audit trail
2. Quality system audits
3. Standard operating procedures (SOPs)
4. Temperature monitoring systems
5. Equipment maintenance schedule
6. Log books
7. Staff training and proficiency testing

Importance:

- To obtain accurate results.
- To obtain reproducibility in pre-analytical factors.
- To facilitate standardization
- To be efficient
- To identify potential problems and to have a record of how to troubleshoot recurring issues
- To facilitate communication between different users of the instrumentation

What is fit for purpose?

TMIC
The Metabolomics Innovation Centre

TMIC (Wishart Node) analytical services:

- We are an ISO-compliant lab: ISO 17025 certification (completed in 2021) with ISO 15189 certification expected in June 2023
- All assays meet standard ISO 17025/15189 compliance and measurement criteria with extensive documentation on CVs, SDs, precision, inter-day variation, intra-day variation, trueness, LOD, LOQ, etc. for every analyte

Quality Software: In-house developed electronic quality management system (eQMS)

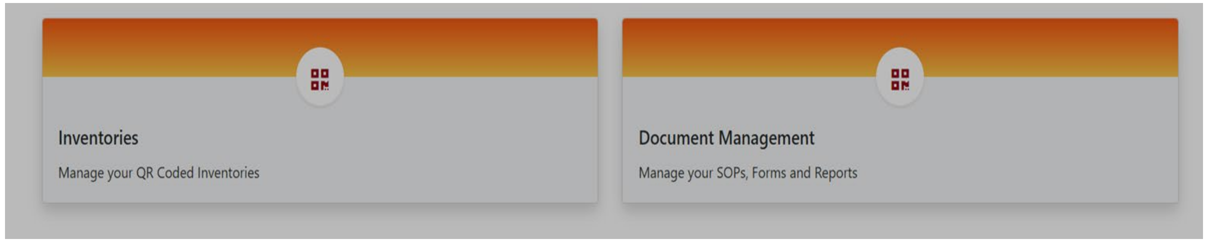

Quality Tool (Plan-Do-Check-Act (PDCA)

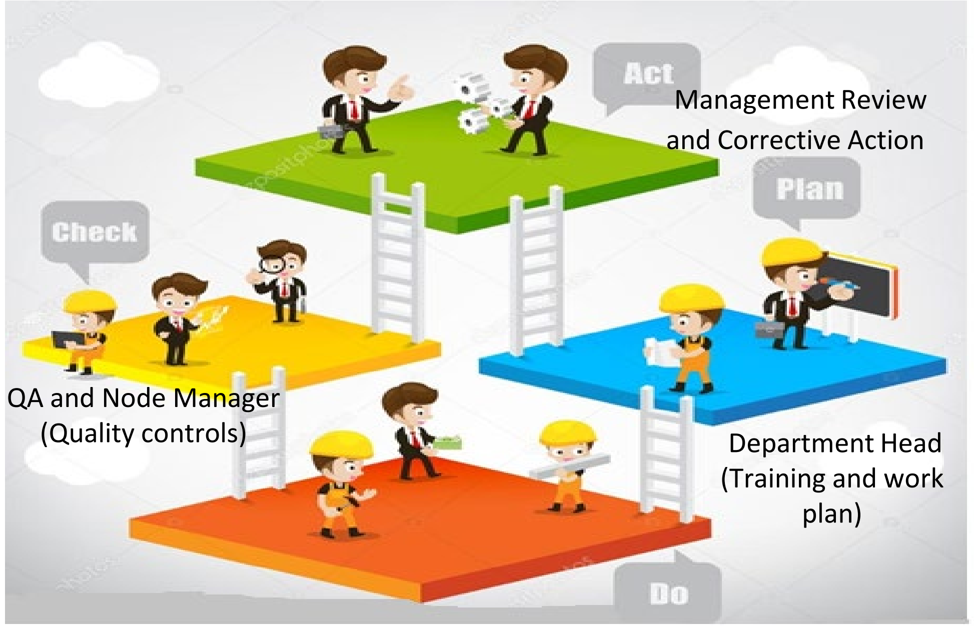

Quality Software: In-house developed electronic quality management system (eQMS)

What is fit for purpose?

BASF Metabolome Solutions (BMS)

■ Additional QA measures established

- Staff training records
- Lab audits and SOPs (or work instructions)
- Instrument validation
- Performance monitoring and maintenance documentation
- Controlled storage (temperature, location)
- Process automation & LIMS
  - → Instrument, day-to-day and inter-study comparability required for long-term projects
    - MetaMap®Tox Metabolome Database
    - Regulatory use for read-across under REACH
    - Herbicide mode-of-action studies

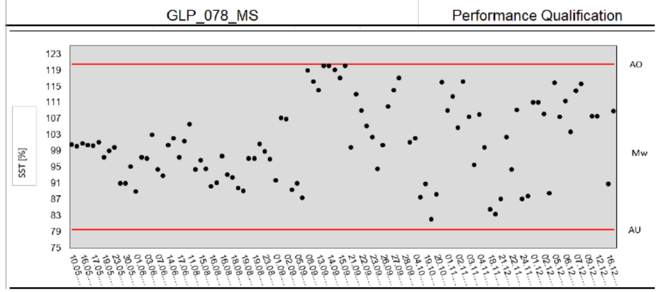

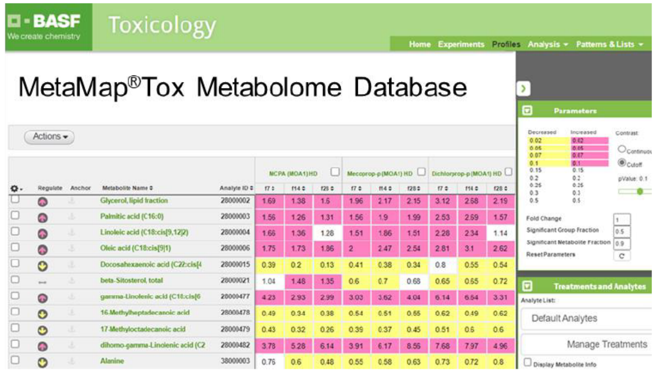

BASF
We create chemistry

2

Oliver Schmitz & Michael Herold,
BASF Metabolome Solutions

Metabolomics Quality Assurance & Quality Control Consortium (mQACC)

Log Books

Electronic Notebooks

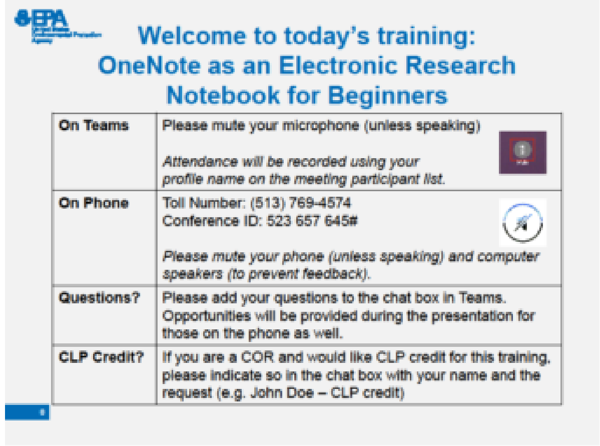

Welcome to today's training: OneNote as an Electronic Research Notebook for Beginners

On Teams	Please mute your microphone (unless speaking)<br>*Attendance will be recorded using your profile name on the meeting participant list.*
On Phone	Toll Number: (513) 769-4574<br>Conference ID: 523 657 645#<br>*Please mute your phone (unless speaking) and computer speakers (to prevent feedback).*
Questions?	Please add your questions to the chat box in Teams. Opportunities will be provided during the presentation for those on the phone as well.
CLP Credit?	If you are a COR and would like CLP credit for this training, please indicate so in the chat box with your name and the request (e.g. John Doe – CLP credit)

- Microsoft OneNote currently implemented for electronic logbooks, notebooks, etc
- Image to the left is EPA's internal training presentation that contains generic examples (open embedded PDF, if curious)
- Several example pages from my lab follow in various applications (Log books, etc.)

Jonathan Mosley, EPA

Equipment Maintenance

Electronic Notebook – Scheduled Maintenance Checklist

- Located in electronic notebook on instrument PC
- References SOP for easy access to maintenance procedures
- Easily updated during/after maintenance procedure
- Duplicate entry kept in paper log book for each instrument

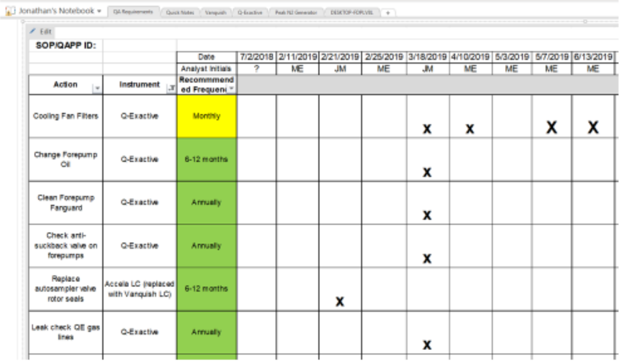

Jonathan's Notebook | QA Requirements | Quick Notes | Vanquish | Q-Exactive | Peak N2 Generator | DESKTOP-FDPLVIL

Edit

SOP/QAPP ID:

Action	Instrument	Date / Analyst Initials / Recommended Frequency	7/2/2018	2/11/2019	2/21/2019	2/25/2019	3/18/2019	4/10/2019	5/3/2019	5/7/2019	6/13/2019
		Analyst Initials	?	ME	JM	ME	JM	ME	ME	ME	ME
Cooling Fan Filters	Q-Exactive	Monthly					x	x		X	X
Change Forepump Oil	Q-Exactive	6-12 months					x				
Clean Forepump Fanguard	Q-Exactive	Annually					x				
Check anti-suckback valve on forepumps	Q-Exactive	Annually					x				
Replace autosampler valve rotor seals	Accela LC (replaced with Vanquish LC)	6-12 months			x						
Leak check QE gas lines	Q-Exactive	Annually					x				

Jonathan Mosley, EPA

Electronic Notebook– Pump pressure log sheet

- Located in electronic notebook on instrument PC
- Keeps daily record of pump pressures to monitor performance of LC pumps across studies
- Easily updated during priming of LC system
- Location in Electronic Notebook allows easy access to SOP for relevant procedures

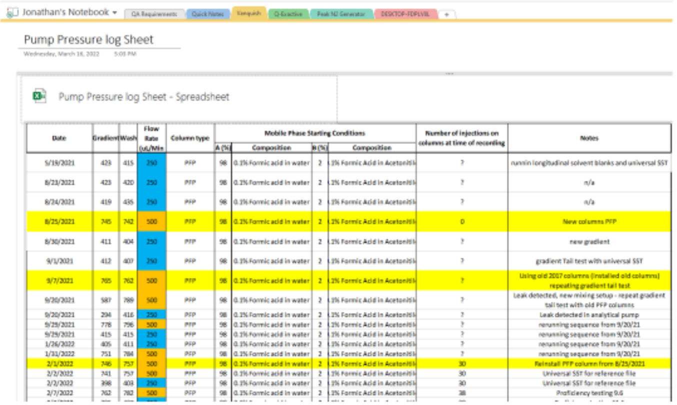

Jonathan's Notebook | QA Requirements | Quick Notes | Vanquish | Q-Exactive | Peak N2 Generator | DESKTOP-FDPLVIL

Pump Pressure log Sheet

Wednesday, March 16, 2022 5:03 PM

Pump Pressure log Sheet - Spreadsheet

Date	Gradient	Wash	Flow Rate (uL/Min)	Column type	Mobile Phase Starting Conditions: A (%)	Composition	B (%)	Composition	Number of injections on columns at time of recording	Notes
5/19/2021	423	415	250	PFP	98	0.1% Formic acid in water	2	1% Formic Acid in Acetonitil	?	runnin longitudinal solvent blanks and universal SST
8/23/2021	423	420	250	PFP	98	0.1% Formic acid in water	2	1% Formic Acid in Acetonitil	?	n/a
8/24/2021	419	435	250	PFP	98	0.1% Formic acid in water	2	1% Formic Acid in Acetonitil	?	n/a
8/25/2021	745	742	500	PFP	98	0.1% Formic acid in water	2	1% Formic Acid in Acetonitil	0	New columns PFP
8/30/2021	411	404	250	PFP	98	0.1% Formic acid in water	2	1% Formic Acid in Acetonitil	?	new gradient
9/1/2021	412	407	250	PFP	98	0.1% Formic acid in water	2	1% Formic Acid in Acetonitil	?	gradient Tail test with universal SST
9/7/2021	765	762	500	PFP	98	0.1% Formic acid in water	2	1% Formic Acid in Acetonitil	?	Using old 2017 columns (installed old columns) repeating gradient tail test
9/20/2021	587	789	500	PFP	98	0.1% Formic acid in water	2	1% Formic Acid in Acetonitil	?	Leak detected, new mixing setup - repeat gradient tail test with old PFP columns
9/20/2021	294	416	250	PFP	98	0.1% Formic acid in water	2	1% Formic Acid in Acetonitil	?	Leak detected in analytical pump
9/29/2021	778	796	500	PFP	98	0.1% Formic acid in water	2	1% Formic Acid in Acetonitil	?	rerunning sequence from 9/20/21
9/29/2021	415	415	250	PFP	98	0.1% Formic acid in water	2	1% Formic Acid in Acetonitil	?	rerunning sequence from 9/20/21
1/26/2022	405	411	250	PFP	98	0.1% Formic acid in water	2	1% Formic Acid in Acetonitil	?	rerunning sequence from 9/20/21
1/31/2022	751	784	500	PFP	98	0.1% Formic acid in water	2	1% Formic Acid in Acetonitil	?	rerunning sequence from 9/20/21
2/1/2022	746	757	500	PFP	98	0.1% Formic acid in water	2	1% Formic Acid in Acetonitil	30	Reinstall PFP column from 8/25/2021
2/2/2022	741	757	500	PFP	98	0.1% Formic acid in water	2	1% Formic Acid in Acetonitil	30	Universal SST for reference file
2/2/2022	398	403	250	PFP	98	0.1% Formic acid in water	2	1% Formic Acid in Acetonitil	30	Universal SST for reference file
2/7/2022	762	782	500	PFP	98	0.1% Formic acid in water	2	1% Formic Acid in Acetonitil	38	Proficiency testing 9.6
[illegible]	[illegible]	[illegible]	[illegible]	[illegible]	[illegible]	[illegible]	[illegible]	[illegible]	[illegible]	[illegible]

Jonathan Mosley, EPA

Metabolomics Quality Assurance & Quality Control Consortium (mQACC)

Temperature Monitoring

Freezer monitoring

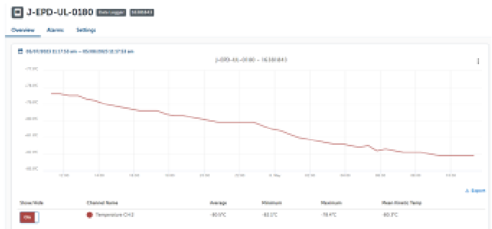

JMosley-Metabolomics-Elect... | QA Requirements | Project Boards | Galileo Access | Computer Troubleshoot

Freezer Locations

Monday, October 04, 2021 2:29 PM

All freezer locations related to this research effort can be found at the following location(s):

LSASD, Room E-107 (South Wall):

A010 (J-EPD-UL-180)

Make: Thermo Scientific

Model: 903

S/N: 835738-378

EPA Decal: B04411

SES ID: 010227

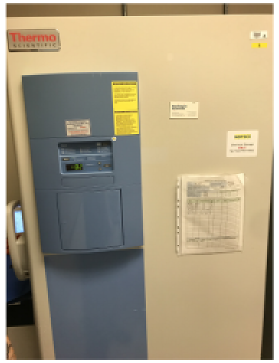

- Use DicksonOne (external contractor) for freezer temperature monitoring
- Alerts sent via email/SMS when out of acceptable range
- Freezer locations/status tracked in Electronic Notebook

Jonathan Mosley, EPA

TIC fluctuations due to lab temp changes

Laboratory temperature spikes

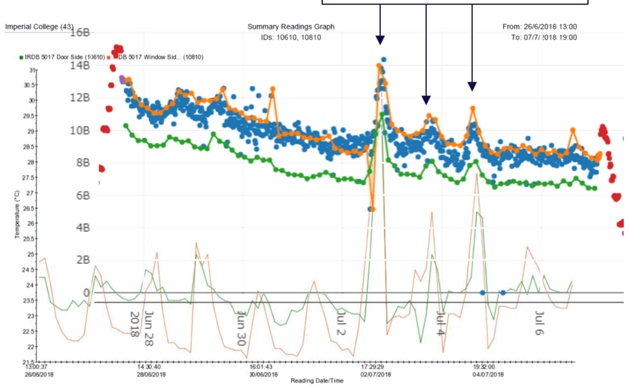

HILIC +ESI MS total ion count (TIC)

- Study samples
- Study reference (SR)
- SR dilution series
- Long term reference (LTR)

T-Scan graphs for the probes nearest the instrument used during data acquisition

- Probe close to the lab door
- Probe close to the lab window

6

Pantelis Takis, National Phenome Centre

Metabolomics Quality Assurance & Quality Control Consortium (mQACC)

Standard Operating Procedures

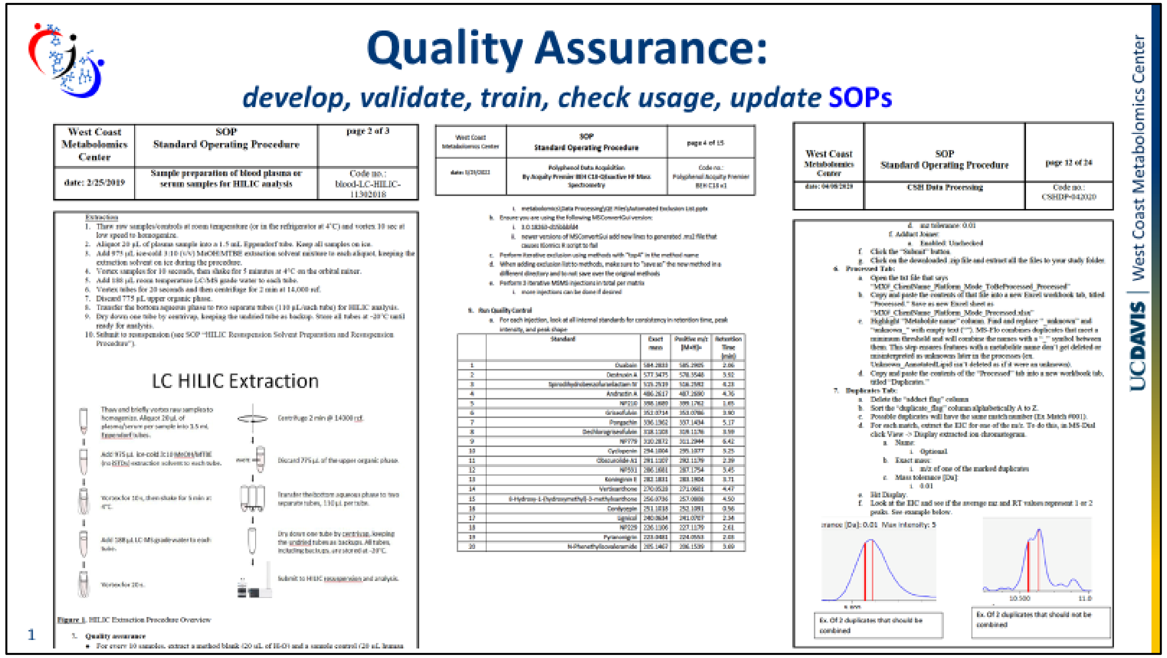

Fiehn lab, UC Davis

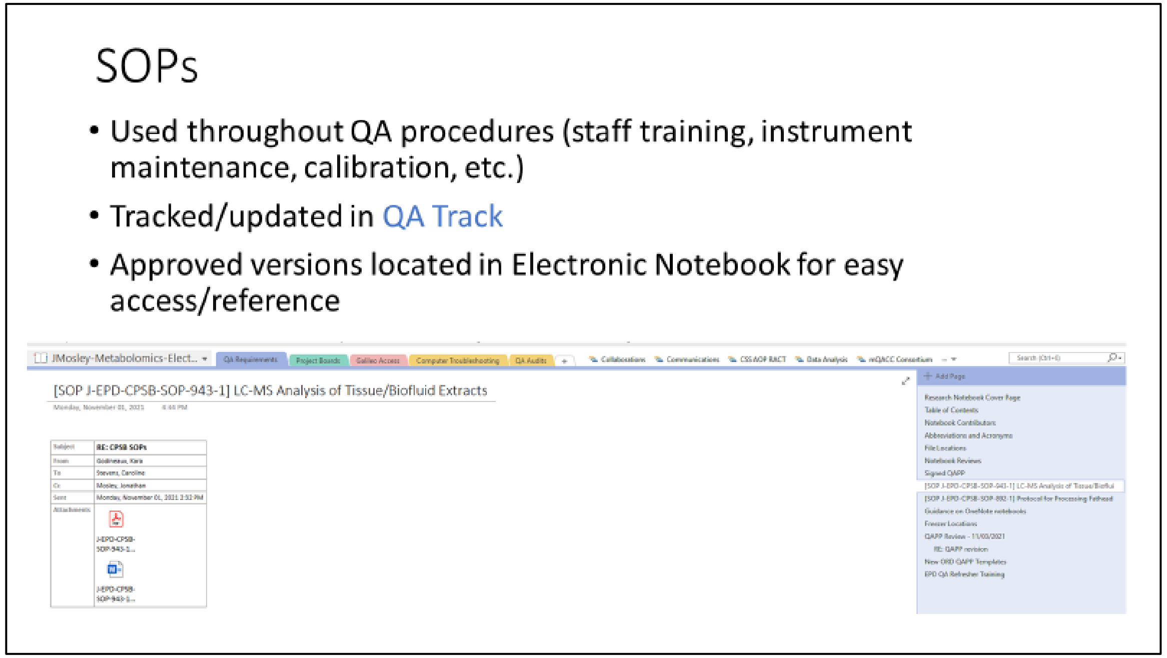

Jonathan Mosley, EPA

Standard Operating Procedures

Metabolomics Quality Assurance & Quality Control Consortium (mQACC)

Pantelis Takis, National Phenome Centre

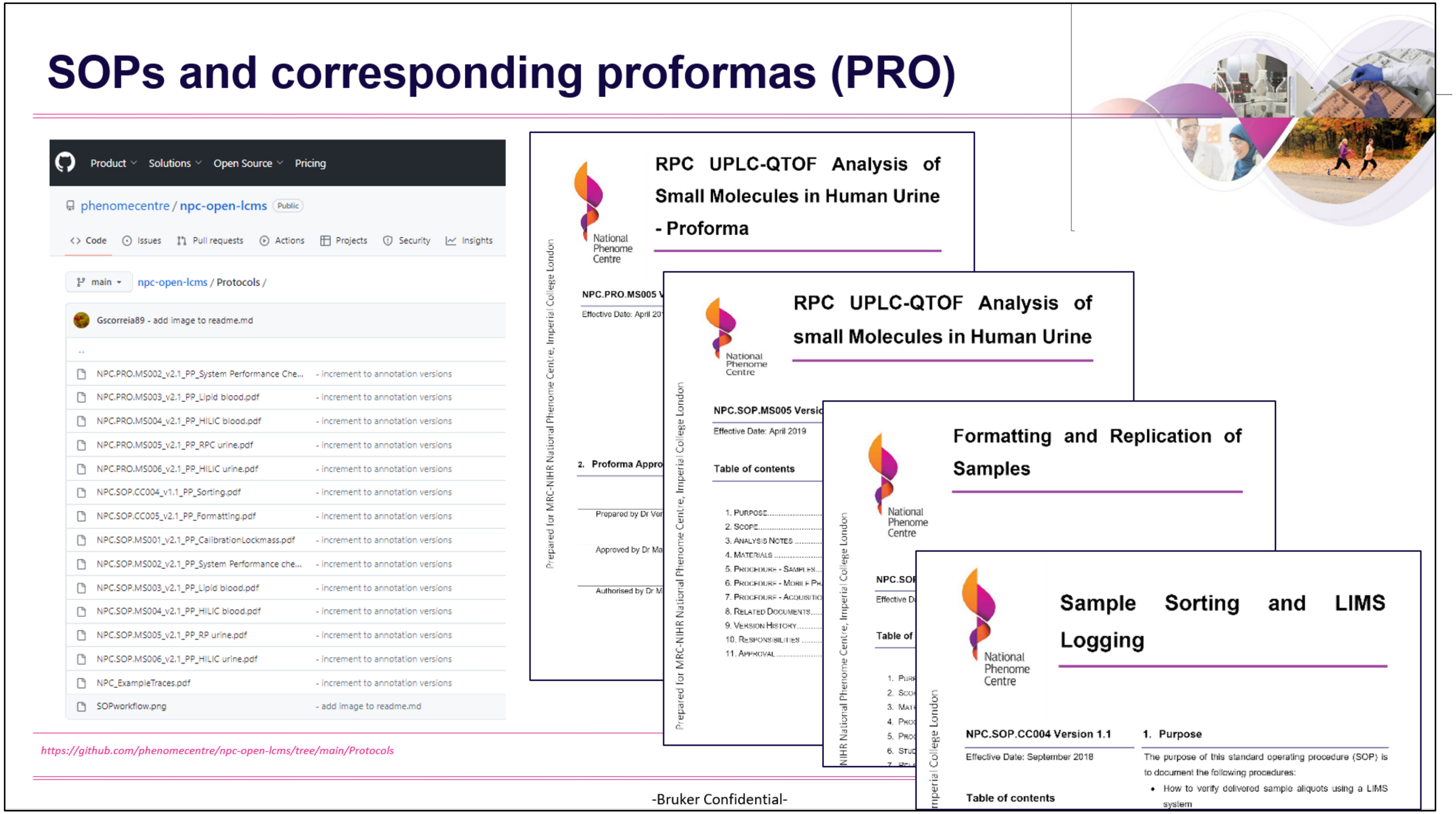

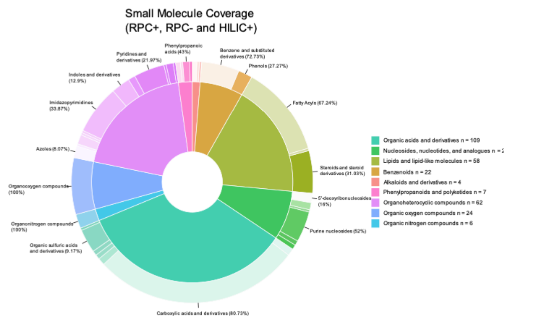

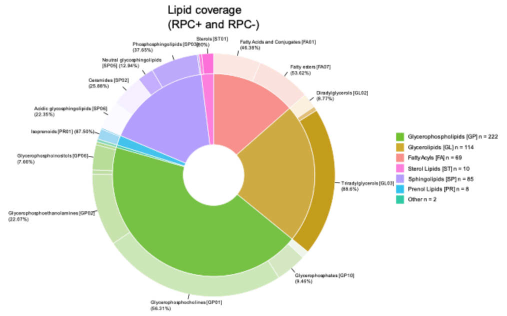

LC-MS Open Platform

- LC-MS protocols and methods
- Annotations library (>700 small molecule metabolites and lipid species)
- Open-source software tools
- Training material

Proficiency Testing

Metabolomics Quality Assurance & Quality Control Consortium (mQACC)

Proficiency/Competency Testing in Untargeted Analyses

What is it?

A set of experiments used to demonstrate that an analyst has the necessary skills to execute sample analysis that meets your quality metrics.

Example analyses:

1) Low lift – Prepare and analyze a batch of biological technical replicates and blanks. Assess data for LC and MS precision, carryover, and sensitivity. *Sensitivity can be determined by establishing a set of low-level endogenous markers from the biological samples that are detectable when the system is running well.*
2) Medium lift – Same as above but add biological QC samples. Biological QC samples are a set of samples with a known biological signature that can be assessed, for example, diabetic vs non-diabetic samples, or fed vs fasted samples. Assess data as described above with addition of detection of the specific biological signature.
3) Best – Same as above, but do not notify personnel that they are being tested. Submit blinded samples into queue for analysis and after QC by analyst is complete, have the data reviewed in full by QA and Lab Director to determine pass/fail of proficiency. Document and store testing outcomes in employee's record.

Note: proficiency/competency testing should include all aspects of sample analysis and quality control review. Examples include preparation of mobile phases and any other solutions needed in sample preparation and analysis, installation of new column and column conditioning as appropriate, as well as how well the analyst reviews the data for quality metrics.

How often?

Upon hire, 6 months later, then yearly

Note: If an analyst has more than 2 QC failures that can be attributed to user error, retraining and proficiency testing starts from the beginning

© 2023 Metabolon, Inc. All rights reserved 2

Annie Evans, Metabolon

Documentation & Audit Trail

Metabolomics Quality Assurance & Quality Control Consortium (mQACC)

Annie Evans, Metabolon

Audit Trail

What is it?

The ability to track exactly what was done at any given time point and my whom throughout the entire process.

What needs to have an audit trail?

Ideally, every aspect of a project should have documentation or software logs detailing who did something, when they did it and in some cases why they did it. A few examples include: Who received the package and when? Who accessioned the samples and when, were there any issues noted during accessioning? Who put the samples in what freezer, when? Who made the solvents, when and what lots of reagents were used to make those solvents? Who created the plate layout, when, and what samples were present? Who performed instrument QC review, what did they find and when? Who changed the peak integration and when? Who sent the final report to the investigator, when and what files were sent?

What is it for?

An audit trail is an important tool in troubleshooting issues, but also in defending and validating the work performed. Extensive audit trails are required in regulated environments, particularly in clinical environments where audit trails often also require e-signatures and a written explanation of why any change was made throughout the process.

© 2023 Metabolon, Inc. All rights reserved

4

Jonathan Mosley, EPA

QA Documentation

- EPA/ORD uses web-based app QA Track for implementing QA for environmental data
- Quality Assurance Project Plan (QAPP) required for all projects before data is collected
- QA manager reviews every product (presentations/publications containing EPA data) against QAPP prior to dissemination

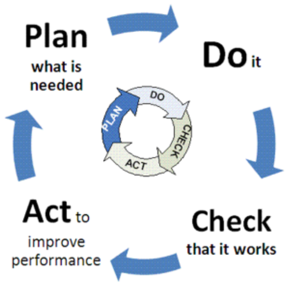

Required according to EPA Policy and Program Requirements for the Mandatory Agency-Wide Quality System (CIO 2105.0)

Quality System Audits

Metabolomics Quality Assurance & Quality Control Consortium (mQACC)

Quality System Audits

What is it?
A systematic review of all systems and processes in place to support sample analysis. Audits are conducted by an unbiased individual not involved in day-to-day operations looking for compliance to SOPs, reviewing nonconformances, and noting needed areas of improvement.

What is Covered?
An auditor reviews the processes and systems, usually established in the form of SOPs, that a laboratory has in place. This involves interviews with staff, watching staff as they perform their tasks, and reviewing that required documentation is present and completed accurately. An auditor will also review the log of nonconformances such as quality events, deviations, and CAPAs to look for trends in where issues occur, perform a root cause analysis and use this information to recommend areas of improvement.

Ideally, every activity occurring before, during and after sample analysis is audited, examples including logging potential projects, shipping/receiving activities, sample storage, sample preparation, data acquisition, QC reviews, statistics, report generation, report delivery, staff training and proficiency status, data security and storage, etc.

How often?
The frequency of audits depends on level of regulation required:
1) Clinical testing requires that the entire system be audited yearly (pre-sample analysis, sample analysis and post-sample analysis)
2) RUO testing requires that the entire system be audited at a minimum of every two years *(note that it is acceptable to break this into subparts and audit overtime (audit one part of the process per month) as long as every part of the process is audited every two years)*

© 2023 Metabolon, Inc. All rights reserved
3

Annie Evans, Metabolon

QA Documentation

- QAPP must be reviewed annually
- External audits every 3 years

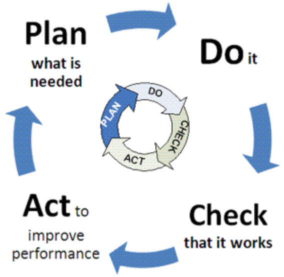

Required according to EPA Policy and Program Requirements for the Mandatory Agency-Wide Quality System (CIO 2105.0)

Jonathan Mosley, EPA

Metabolomics Quality Assurance & Quality Control Consortium (mQACC)

Conclusions

- QA systems *assure data quality*
- Extent of QC should be fit for intended purpose and appropriately scaled to lab environment and activites
- Simple, enabling systems are key for user compliance

Want to get involved? mQACC has recently launched the QA task group!

Chairs
Golla, Srujana kolasrujana@gmail.com
Johns Hopkins Medicine

Rafea Naffa Rafea.Naffa@fonterra.com
Fonterra Co-operative Group Limited

Vice chairs
Álvaro Fernández Ochoa
alvaro.fernandezochoa@mdc-berlin.de
Berlin Institute of Health

Shaowei An anshaowei@westlake.edu.cn
Westlake University

Thanks to the following mQACC members representing the following organisations:

- Oliver Fiehn, UC Davis
- Sindhu Nair, TMIC
- Oliver Schmitz & Michael Herold, BASF Metabolome Solutions
- Panteleimon Takis, National Phenome Centre
- María Eugenia Monge, CONICET at CIBION
- Oliver Schmitz & Michael Herold, BASF Metabolome Solutions
- Annie Evans, Metabolon
- Jonathan Mosley, EPA

Polling Question

Q5 Guidance for quality assurance in untargeted LC-MS-based metabolomics studies in upcoming mQACC guidelines should cover (choose all that apply):

a. Considerations in adopting QA practices that are “fit-for-purpose”
b. Descriptions of tiered QA approaches including use-case scenarios
c. Representative QA templates (e.g., logbooks, protocols)
d. Other
e. I do not think that guidance for this topic should be included
